# Identification of *KIAA0513* and Other Hub Genes Associated With Alzheimer Disease Using Weighted Gene Coexpression Network Analysis

**DOI:** 10.3389/fgene.2020.00981

**Published:** 2020-08-28

**Authors:** Min Zhu, Longfei Jia, Fangyu Li, Jianping Jia

**Affiliations:** ^1^Innovation Center for Neurological Disorders and Department of Neurology, Xuanwu Hospital, Capital Medical University, National Clinical Research Center for Geriatric Diseases, Beijing, China; ^2^Beijing Key Laboratory of Geriatric Cognitive Disorders, Beijing, China; ^3^Clinical Center for Neurodegenerative Disease and Memory Impairment, Capital Medical University, Beijing, China; ^4^Center of Alzheimer’s Disease, Beijing Institute for Brain Disorders, Beijing, China

**Keywords:** Alzheimer disease, dementia, gene expression, hub genes, weighted gene coexpression network analysis

## Abstract

Alzheimer disease (AD) is the most common cause of dementia and creates a significant burden on society. As a result, the investigation of hub genes for the discovery of potential therapeutic targets and candidate biomarkers is warranted. In this study, we used the ComBat method to merge three gene expression datasets of AD from the Gene Expression Omnibus (GEO). During combined analysis, we identified 850 differentially expressed genes (DEGs) from the temporal cortex of AD and cognitively normal (CN) samples. We performed weighted gene coexpression network analysis to build gene coexpression networks incorporating these DEGs to identify key modules and hub genes. We found one module most strongly correlated with AD onset as the key module and 19 hub genes in the key module that were down-regulated in AD brains. According to Gene Ontology and Kyoto Encyclopedia of Genes and Genomes analyses, DEGs were mostly enriched in synapse function, and genes in the key module were mostly related to learning and memory. We selected five little-studied genes, *AP3B2*, *GABRD*, *GPR158*, *KIAA0513*, and *MAL2*, to validate their expression in AD mouse model by performing quantitative real-time polymerase chain reaction. We found that all of them were down-regulated in cortices of 8-month 5xFAD mice compared to those of wild-type mice. We then further investigated their correlations with β-secretase activity and Aβ42 levels in AD samples of different Braak stages. We found that all five hub genes had significant negative associations with β-secretase activity and that *AP3B2* and *KIAA0513* had significant negative associations with Aβ42 levels. We tested the differential expressions of the five hub genes in two AD GEO datasets from the blood and found that *KIAA0513* was significantly up-regulated in patients with both mild cognitive impairment (MCI) and AD and was able to differentiate MCI and AD from CN in the two datasets. In conclusion, these five novel vulnerable genes were involved in AD progression, and KIAA0513 was a promising candidate biomarker for early diagnosis of AD.

## Introduction

Alzheimer disease (AD) is the most common cause of dementia and is manifested as progressive impairments of memory and other cognitive domains. Pathological lesions in AD include β-amyloid (Aβ) plaques, neurofibrillary tangles, synaptic failure, neuronal loss, and brain atrophy (Serrano-Pozo et al., 2011). At present, only *APP*, *PSEN1*, *PSEN2*, and *APOEε4* are considered to be causal genes or variants for AD (Tanzi, 2012; Jia et al., 2020; Neuner et al., 2020). In addition, genome-wide association studies also found AD risk genes including *ABCA7*, *CLU*, *SORL1*, *TREM2*, and so on (Campion et al., 2019; Kunkle et al., 2019). Network analyses of AD-related genes from publications revealed the complexed molecular mechanisms of AD (Hu et al., 2017). *In vitro* studies have identified other genes such as *AChE* (Garcia-Ayllon et al., 2011) and *TFEB* (Guo et al., 2017; Xu et al., 2020) playing roles in AD pathologies, which provides potential targets for AD therapy. However, there are still no effective drugs for AD treatment. Because AD is a complicated disease affected by age, genetic, and environmental factors, a greater number of key genes and their underlying mechanisms must be identified in order to facilitate the discovery of novel therapeutic targets and candidate biomarkers.

Weighted gene coexpression network analysis (WGCNA) is a gene-screening (i.e., ranking) method and a data exploratory tool for finding clusters (i.e., modules) that includes highly correlated genes, which can then be used for identification of candidate biomarkers and therapeutic targets (Langfelder and Horvath, 2008, 2012). This analysis has been applied widely in studies of various diseases, including cancers and neuropsychiatric disorders (Chuang et al., 2017; Radulescu et al., 2018; Ding et al., 2019; Luo et al., 2019). In the current study, we merged three independent gene expression datasets from the Gene Expression Omnibus (GEO) database^1^ using the ComBat method (Johnson et al., 2007) and analyzed the merged datasets to identify differentially expressed genes (DEGs). These DEGs were then used to find key modules and hub genes associated with AD by WGCNA. Gene Ontology (GO) enrichment and Kyoto Encyclopedia of Genes and Genomes (KEGG) pathway analyses were further utilized to identify possible functions of the DEGs and key modules. Finally, five little-characterized hub genes, *AP3B2*, *GABRD*, *GPR158*, *KIAA0513*, and *MAL2*, were chosen to test their expression levels in different Braak stages, their diagnostic values for AD and mild cognitive impairment (MCI), and their correlations with β-secretase activity and Aβ42 levels. Gene Set Enrichment Analysis (GSEA) was used to explore potential biological functions of these hub genes.

## Materials and Methods

### Data Collection

**FIGURE 1:**
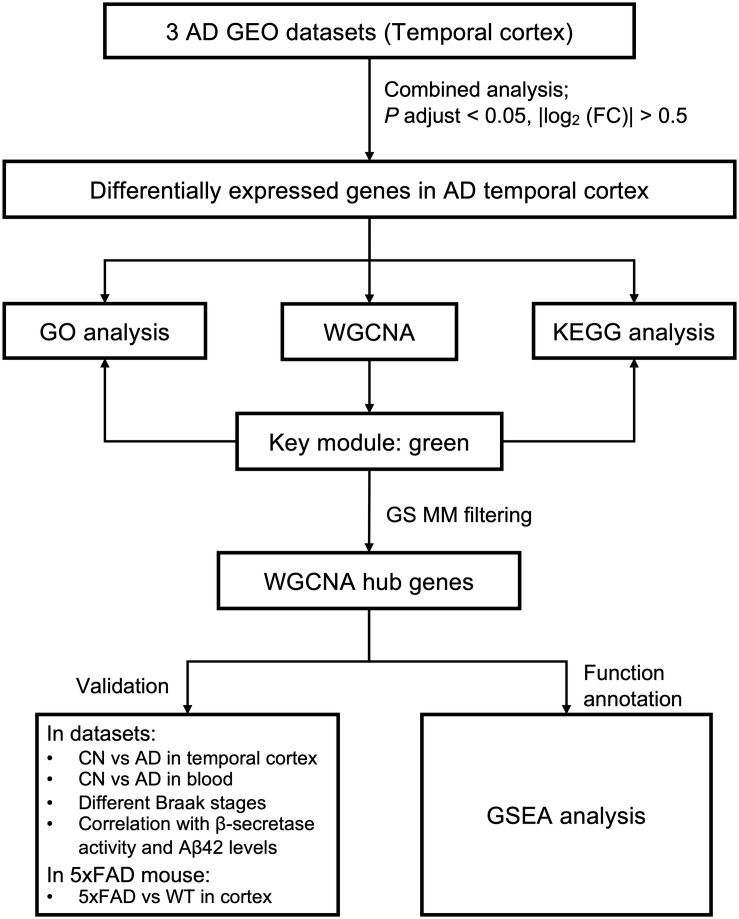
Study workflow. AD, Alzheimer disease; CN, cognitively normal; FC, fold change; GEO, Gene Expression Omnibus; GO, Gene Ontology; GS, gene significance; GSEA, Gene Set Enrichment Analysis; KEGG, Kyoto Encyclopedia of Genes and Genomes; MM, module membership; WGCNA, weighted gene coexpression network analysis; WT, wild type.

Figure 1 shows the overall workflow of this study. All microarray datasets were downloaded from the GEO database^1^. We searched the GEO database for microarray datasets using the keyword “Alzheimer.” Datasets were included if they met the following criteria: (1) were from humans; (2) included expression data from the temporal cortex of both AD and cognitively normal (CN) samples, expression data from the temporal cortex of AD samples with different Braak stages, or blood expression data from AD, MCI, and CN samples; (3) the number of rows in each platform was >30,000; (4) the number of AD samples was ≥10, and the number of CN samples was ≥10; and (5) there were no repeated samples among datasets. Finally, five datasets from the temporal cortex of AD and CN samples; one dataset from the temporal cortex of AD samples with different Braak stages; and two datasets from the blood of AD, MCI, and CN samples were selected. Detailed information for these datasets, including GEO accession ID, dataset country, sample numbers, platform ID, and number of genes in each platform, as well as usage in the current study and references, was recorded and is shown in Table 1. Because GSE132903 had far more samples (98 AD, 97 CN) than the other four datasets from the temporal cortex of AD and CN samples, combining this dataset would have blurred the results of the combined analysis. Thus, GSE132903 (Piras et al., 2019) was used to validate further the differential expression of hub genes. Datasets GSE122063 (28 AD, 22 CN; McKay et al., 2019), GSE36980 (10 AD, 19 CN; Hokama et al., 2014), and GSE5281 (16 AD, 12 CN; Liang et al., 2007, 2008a,b; Readhead et al., 2018) had similar sample sizes and were chosen for the combined analysis in the current study. Dataset GSE118553 (52 AD, 31 CN; Patel et al., 2019) was used to build the coexpression network and to perform GSEA. Dataset GSE106241 (Marttinen et al., 2019) was used to test the expression of hub genes in different Braak stages and to explore the correlation with β-secretase activity and Aβ42 levels. Datasets GSE63060 and GSE63061 (Sood et al., 2015) were used to validate the expression of hub genes in the blood of AD samples. Series matrix files of these datasets and their corresponding platform files were downloaded for the current analysis.

**TABLE 1:** Characteristics of the included datasets.

Tissue	Dataset ID	Country	No. of samples	GPL ID	No. of rows per platform	Usage here	References
Temporal cortex	GSE122063	United States	28 AD, 22 CN	GPL16699	62,976	Combined (ComBat) analysis	McKay et al., 2019
Temporal cortex	GSE36980	Japan	10 AD, 19 CN	GPL6244	33,297	Combined (ComBat) analysis	Hokama et al., 2014
Temporal cortex	GSE5281	United States	16 AD, 12 CN	GPL570	54,675	Combined (ComBat) analysis	Liang et al., 2007, 2008a,b; Readhead et al., 2018
Temporal cortex	GSE118553	United Kingdom	52 AD, 31 CN	GPL10558	48,107	WGCNA, GSEA	Patel et al., 2019
Temporal cortex	GSE132903	United States	98 AD, 97 CN	GPL10558	48,107	Validate diff. expr. of hub genes	Piras et al., 2019
Temporal cortex	GSE106241	Finland	60AD	GPL24170	34,487	Hub expr. for Braak, β-secretase, Aβ42	Marttinen et al., 2019
Blood	GSE63060	United Kingdom	145 AD, 80 MCI, 104 CN	GPL6947	49,576	Hub expr. in blood	Sood et al., 2015
Blood	GSE63061	United Kingdom	139 AD, 110 MCI, 135 CN	GPL10558	48,107	Hub expr. in blood	Sood et al., 2015

*The first three datasets were used for combined analysis, GSE118553 was used for weighted correlation network analysis (WGCNA) and Gene Set Enrichment Analysis (GSEA), and the last four datasets were used for validating the hub genes. AD, Alzheimer disease; CN, cognitively normal; GSE, Gene Expression Omnibus Series; GPL, Gene Expression Omnibus Platform; MCI, mild cognitive impairment.*

### Identification of Differentially Expressed Genes by Combined Analysis

Combined analysis was performed for three datasets, including GSE122063 (McKay et al., 2019), GSE36980 (Hokama et al., 2014), and GSE5281 (Liang et al., 2007, 2008a,b; Readhead et al., 2018). Converting probes to gene symbols for series matrix files of each dataset and merging the gene expression data of these three datasets were conducted using Perl (version 5.18.4) (Wall, 1994, Wall et al., 2000). Batch normalization of the merged file was conducted using the ComBat method from the R package “sva” (Johnson et al., 2007; Leek et al., 2019; R Core Team, 2020). DEG screenings were conducted using the R package “limma” (Ritchie et al., 2015). *P*-values were adjusted using the false discovery rate (FDR) method. Genes with adjusted *P* <0.05 and | log_2_ fold change (FC)| >0.5 were considered as DEGs in the combined analysis. A heatmap of all DEGs was made by the R package “pheatmap” (Kolde, 2019). The R package “OmicCircos” was used to show the chromosomal locations, as well as expression patterns of the top 100 DEGs from the combined analysis.

### Function Enrichment Analyses

GO and KEGG pathway analyses were conducted utilizing the R package “clusterProfiler” (Yu et al., 2012). GO terms and KEGG pathways with adjusted *P* <0.05 were considered to be significant and were exhibited using the R package “GOplot” (Walter et al., 2015).

### Weighted Gene Coexpression Network Analysis

We used the DEGs from the combined analysis to perform WGCNA using expression data from GSE118553 (Patel et al., 2019). The R package “WGCNA” was used to conduct this analysis and to find clinical trait–related modules and hub genes among the DEGs (Langfelder and Horvath, 2008, 2012). To transform the adjacency matrix to a topological overlap matrix, a soft-threshold power with a scale-free *R*^2^ near 0.9 and a slope near 1 was selected. We set the soft-threshold power as 5 (scale-free *R*^2^ = 0.9, slope = -0.96), cut height as 0.25, and the minimal module size as 10 for network construction and module detection. The module with the highest correlation with AD was considered the key module. The network of the key module from WGCNA was generated by Cytoscape (version 3.7.1)^2^. Genes in the key module were selected to perform GO and KEGG analyses to explore their biological functions. Hub genes in WGCNA were defined as those with a gene significance (GS) >0.4 and a module membership (MM) >0.8.

### Validation of Hub Genes in Datasets

Expression data of hub genes extracted from Dataset GSE132903 (Piras et al., 2019) and the other four aforementioned datasets were utilized to validate the differential expression of these hub genes in the temporal cortex. Hub gene expression data, β-secretase activity, and Aβ42 levels in AD samples from GSE106241 (Marttinen et al., 2019) were used to validate the expressions of hub genes in different Braak stages and to investigate their correlations with β-secretase activity and Aβ42 levels. Moreover, GSE63060 and GSE63061 (Sood et al., 2015) were used to explore the differential expressions of hub genes in the blood of AD and MCI samples. Using the two datasets, receiver operating characteristic (ROC) curves were made by GraphPad Prism (version 8.0.0)^3^. Bar plots, box plots, and correlation analysis in this section were all generated using GraphPad Prism (version 8.0.0)^3^.

### Mice

The animal study protocol was reviewed and approved by the Ethics Committee of Capital Medical University. 5xFAD mice express five AD-related mutations in human forms of *APP* and *PSEN1*, including three in *APP* (K670N/M671L, I716V, and V717I) and two in *PSEN1* (M146L and L286V). Eight-month 5xFAD and wild-type (WT, non-transgenic littermates) mice with mixed genders were used in the experiments.

### Quantitative Real-Time Polymerase Chain Reaction

Total RNA was extracted from cortices of 8-month 5xFAD and WT mice by RNAsimple Total RNA Kit (#DP419, TIANGEN, China). RNA 1 μg was used in the following reverse transcription (#RR047, Takara, Japan). Quantitative real-time polymerase chain reaction (qRT-PCR) was done on Applied Biosystems ViiA^TM^ 7 Real-Time PCR System using TB Green^®^ Premix Ex Taq (#RR420, Takara, Japan). β-Actin was used as internal control, and relative expression was determined using 2^–ΔΔCT^ method. Primers were designed and synthesized by BGI Tech Solutions (Beijing Liu He) Co., Ltd. Sequences of primers were shown in Supplementary Table 1.

### Gene Set Enrichment Analysis

GSEA (version 4.0.3, Broad Institute, Inc., Massachusetts Institute of Technology, and Regents of the University of California) was conducted to explore the possible biological functions of the hub genes (Mootha et al., 2003; Subramanian et al., 2005). AD samples in GSE118553 (Patel et al., 2019) were divided into two groups according to the expression level of the hub genes. The database “c2.cp.kegg.v7.0.symbols.gmt” was chosen for enrichment. Terms with *P* <0.05 and FDR <0.25 were defined as significant.

### Statistical Analysis

The normality test and homogeneity of variance test were performed on data extracted from GEO datasets. Data that passed these two tests then underwent *t*-testing for comparisons between two groups and analysis of variance (ANOVA) testing for comparisons among three or more groups. After ANOVA analysis, a Dunnett multiple-comparisons test was used for *post-hoc* testing. Data that passed the normality test, but did not pass the homogeneity of variance test, underwent *t*-testing with Welch correction for comparisons between two groups and the Brown–Forsythe ANOVA test for comparisons among three or more groups. Data that did not pass the normality test underwent non-parametric testing, using the Kruskal–Wallis test and Dunn multiple-comparisons test for comparisons among three or more groups. GraphPad Prism (version 8.0.0) (see text footnote 4) was utilized to perform the above tests.

## Results

### Screening of DEGs by Combined Analysis

**FIGURE 2:**
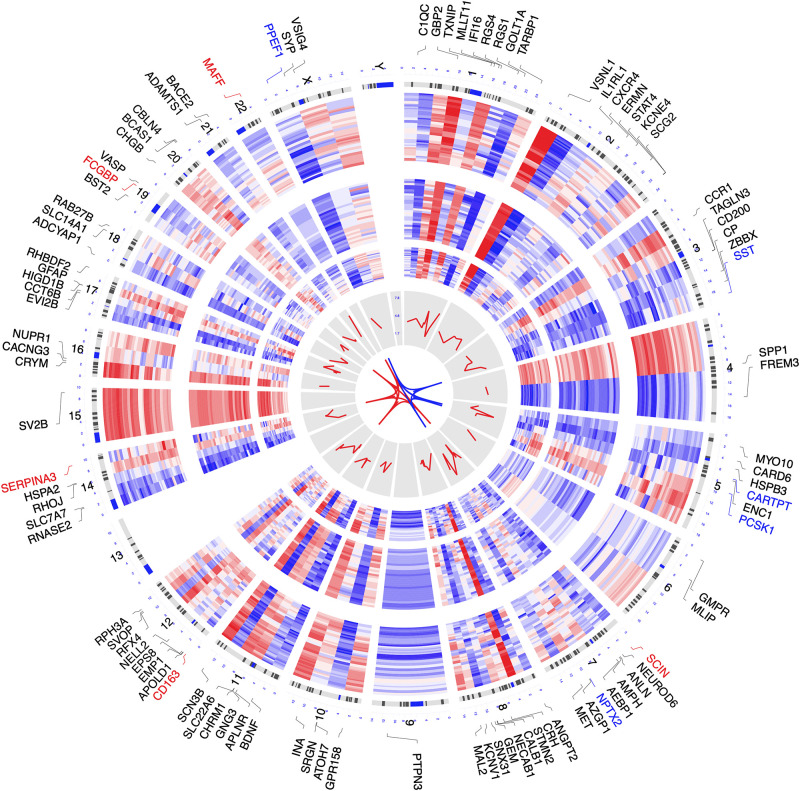
Circos plot of expression patterns and chromosomal positions of top 100 differentially expressed genes (DEGs). The outer circle represents chromosomes, and lines coming from each gene point to their specific chromosomal locations. The three Alzheimer disease (AD) microarray datasets from Gene Expression Omnibus (GEO) used for combined analysis are represented in the inner circular heatmaps. GSE122063, GSE36980, and GSE5281 are presented from the outside to the inside. The red lines in the inner layer indicate -log_10_ (adjusted *P*-value) of each gene. According to |log_2_ fold change|, the top five up-regulated genes (red) and the top five down-regulated genes (blue) are connected with red and blue lines in the center of the Circos plot.

Datasets GSE122063 (McKay et al., 2019), GSE36980 (Hokama et al., 2014), and GSE5281 (Liang et al., 2007, 2008a,b; Readhead et al., 2018) were included in the combined analysis. After combined analysis, 850 DEGs (223 up-regulated and 627 down-regulated) were identified and are shown in heatmap and volcano plots (Supplementary Figures 1, 2 and Supplementary Table 2). The polarity of genes described as “up-regulated” or “down-regulated” in this article is with respect to AD vs. CN. The top 100 DEGs (according to | log_2_FC|, including top 50 up-regulated genes, as well as top 50 down-regulated genes) were chosen to visualize their chromosomal locations and expression patterns across the three datasets used for combined analysis, as well as their logarithmic adjusted *P*-values shown in the inner layer (Figure 2). The top five up-regulated genes were *SERPINA3*, *FCGBP*, *MAFF*, *SCIN*, and *CD163*, whereas the top five down-regulated genes were *CARTPT*, *SST*, *PCSK1*, *PPEF1*, and *NPTX2* (Figure 2).

### Functional Enrichment Analysis of DEGs

**FIGURE 3:**
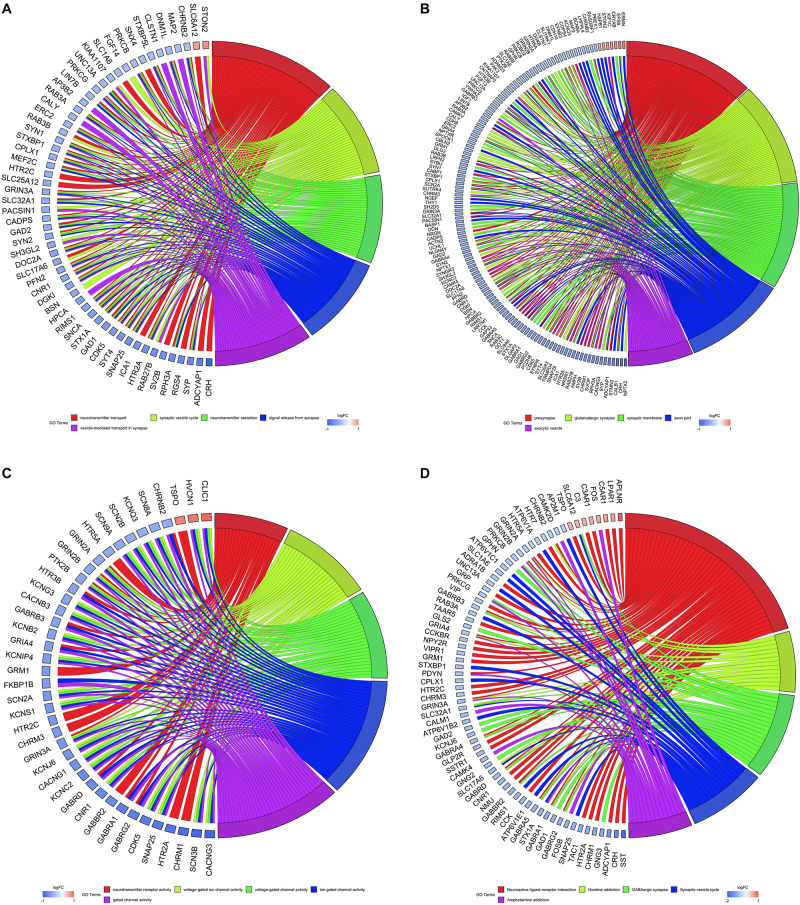
Gene Ontology (GO) and Kyoto Encyclopedia of Genes and Genomes (KEGG) analyses of all differentially expressed genes. Chord plots indicate enrichment analysis of genes. **(A)** Biological process of GO analysis. **(B)** Cellular component of GO analysis. **(C)** Molecular function of GO analysis. **(D)** KEGG pathways.

All DEGs were utilized to perform GO and KEGG analyses, and the top five of these terms based on their adjusted *P*-values are shown in chord plots (Figure 3). We found enrichments in several biological process terms for GO analysis. The top five terms were neurotransmitter transport, synaptic vesicle cycle, neurotransmitter secretion, signal release from synapse, and vesicle-mediated transport in synapse, which are shown in Figure 3A. The top five cellular component terms for GO analysis were presynapse, glutamatergic synapse, synaptic membrane, axon part, and exocytic vesicle (Figure 3B). The top five molecular function terms for GO analysis were neurotransmitter receptor activity, voltage-gated ion channel activity, voltage-gated channel activity, ion gated channel activity, and gated channel activity (Figure 3C). For KEGG pathway analysis, DEGs were mostly enriched in neuroactive ligand-receptor interaction, nicotine addiction, GABAergic synapse, synaptic vesicle cycle, and amphetamine addiction pathways (Figure 3D). Further, we did enrichment analyses in up-regulated genes and down-regulated genes of AD brains separately and found that up-regulated genes were mostly enriched in regulation of angiogenesis, whereas down-regulated genes were mostly related to synaptic functions (Supplementary Figures 3, 4).

### WGCNA and Key Module Identification

**FIGURE 4:**
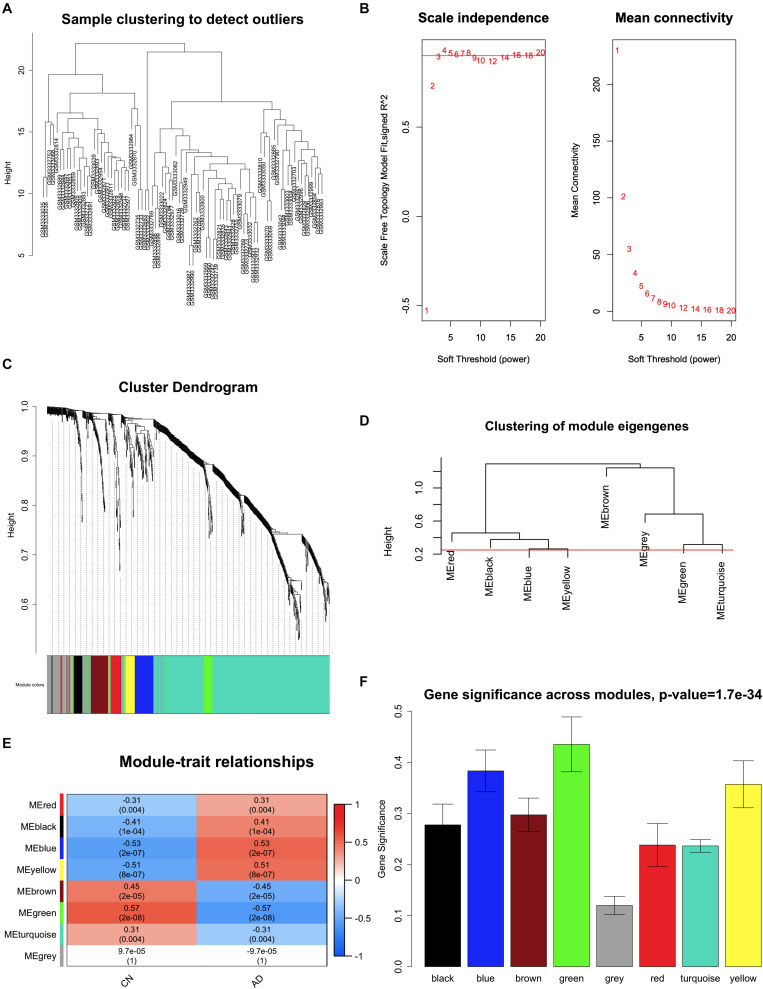
Key module correlated with Alzheimer disease identified by weighted gene coexpression network analysis (WGCNA). **(A)** Clustering of samples to detect outliers. **(B)** Scale-free topology model (left) and mean connectivity (right) for finding the soft-thresholding power. The power selected is 5. **(C)** Cluster dendrogram of genes. **(D)** Clustering of all modules. The red line indicates the height cutoff (0.25). **(E)** Heatmap shows the relationships between different modules and clinical traits. **(F)** Gene significance in different modules associated with AD. AD, Alzheimer disease; CN, cognitively normal.

**FIGURE 5:**
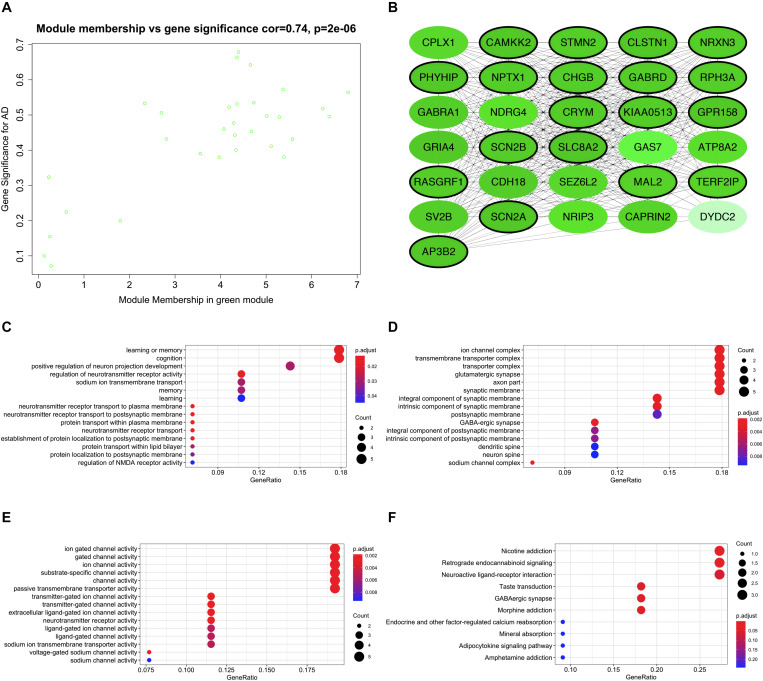
Functional enrichment of the key module. **(A)** Scatter plot of module eigengenes in the key module. **(B)** Interaction network of genes in the key module. Genes in black circles are hub genes in the key module. **(C)** Bubble plots of biological process of gene ontology (GO) analysis. **(D)** Bubble plots of cellular component of GO analysis. **(E)** Bubble plots of molecular function of GO analysis. **(F)** Bubble plots of KEGG pathways.

Expression data of 823 DEGs were extracted from GSE118553 (27 DEGs not available; Patel et al., 2019) and used to conduct WGCNA (Figure 4). By setting the soft-threshold power as 5 (scale-free *R*^2^ = 0.9, slope = -0.96; Figure 4B and Supplementary Figure 5) and cut height as 0.25, we acquired eight modules (Figures 4C,D), with non-clustering DEGs in the gray module. Genes in each module are shown in Supplementary Table 3. From the heatmap of module–trait correlations, we found that the green module was the most highly correlated with AD (correlation coefficient = -0.57, *P* = 2 × 10^–8^; Figures 4E,F). We also found that the blue, yellow, and brown modules had similar correlation coefficients with the green module. However, after comparing the relationships between GS and MM, we considered the green module (correlation coefficient = 0.74, *P* = 2 × 10^–6^; Figure 5A and Supplementary Figure 6) to be the key module associated with AD and therefore analyzed it further in detail. The key module contained 31 genes (Figure 5A and Supplementary Table 3), and the interaction of the genes in the key module is shown in Figure 5B. To explore the potential biological functions of genes in the key module, GO and KEGG analyses were performed, and the enrichments are shown in Figures 5C–F. The results indicated that genes in the key module were mostly related to biological processes like learning or memory and cognition (Figure 5C) and enriched in pathways like nicotine addiction and retrograde endocannabinoid signaling (Figure 5F). Under the threshold of *MM* >0.8 and *GS* >0.4, we identified the following 19 down-regulated hub genes in the key module: *AP3B2*, *CAMKK2, CHGB*, *CLSTN1*, *CRYM*, *GABRD*, *GPR158*, *KIAA0513*, *MAL2*, *NPTX1*, *NRXN3*, *PHYHIP*, *RASGRF1*, *RPH3A*, *SCN2A*, *SCN2B*, *SLC8A2*, *STMN2*, and *TERF2IP*.

### Validation of the Expression of Hub Genes

**FIGURE 6:**
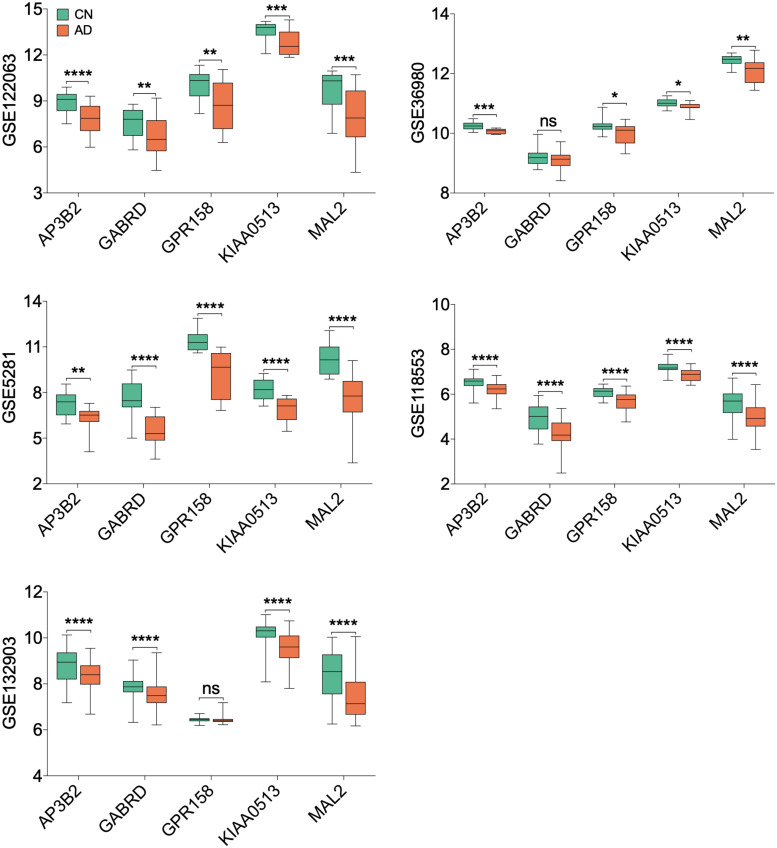
Expression of five hub genes in the temporal cortex. Only hub genes that have been little characterized to be associated with Alzheimer disease (AD) are listed. The green box indicates the cognitively normal group, and the orange box indicates the AD group. *T*-testing was performed to compare the means of two groups. *P < 0.05; **P < 0.01; ***P < 0.001; ****P < 0.0001. AD, Alzheimer disease; CN, cognitively normal; ns, no significance.

**FIGURE 7:**
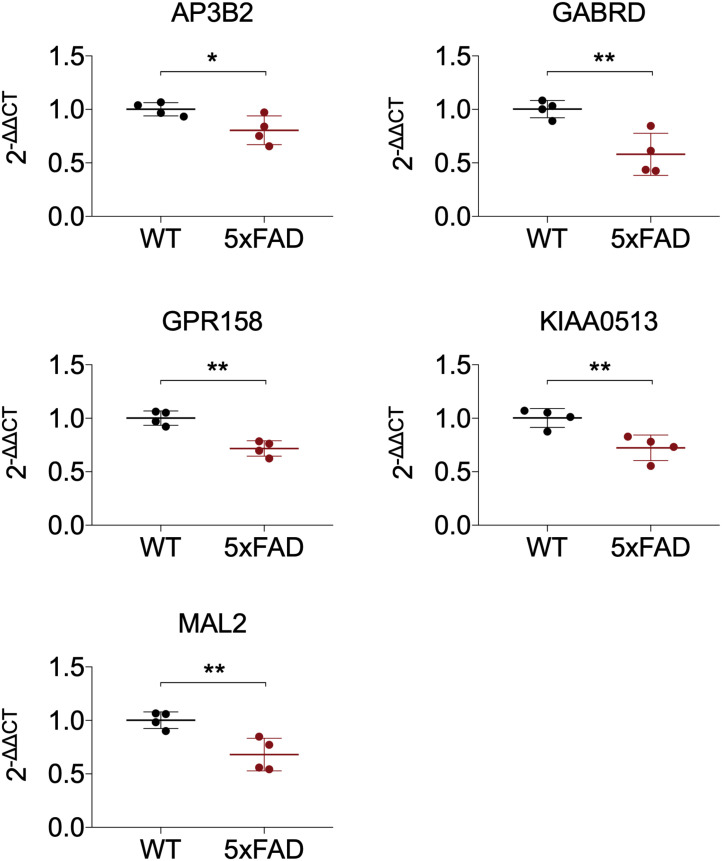
Expression of five hub genes in cortex of 5xFAD mice. The results were presented as mean ± standard deviation (*t*-testing; *n* = 4 in each group). *P < 0.05; **P < 0.01. WT, wild type.

**FIGURE 8:**
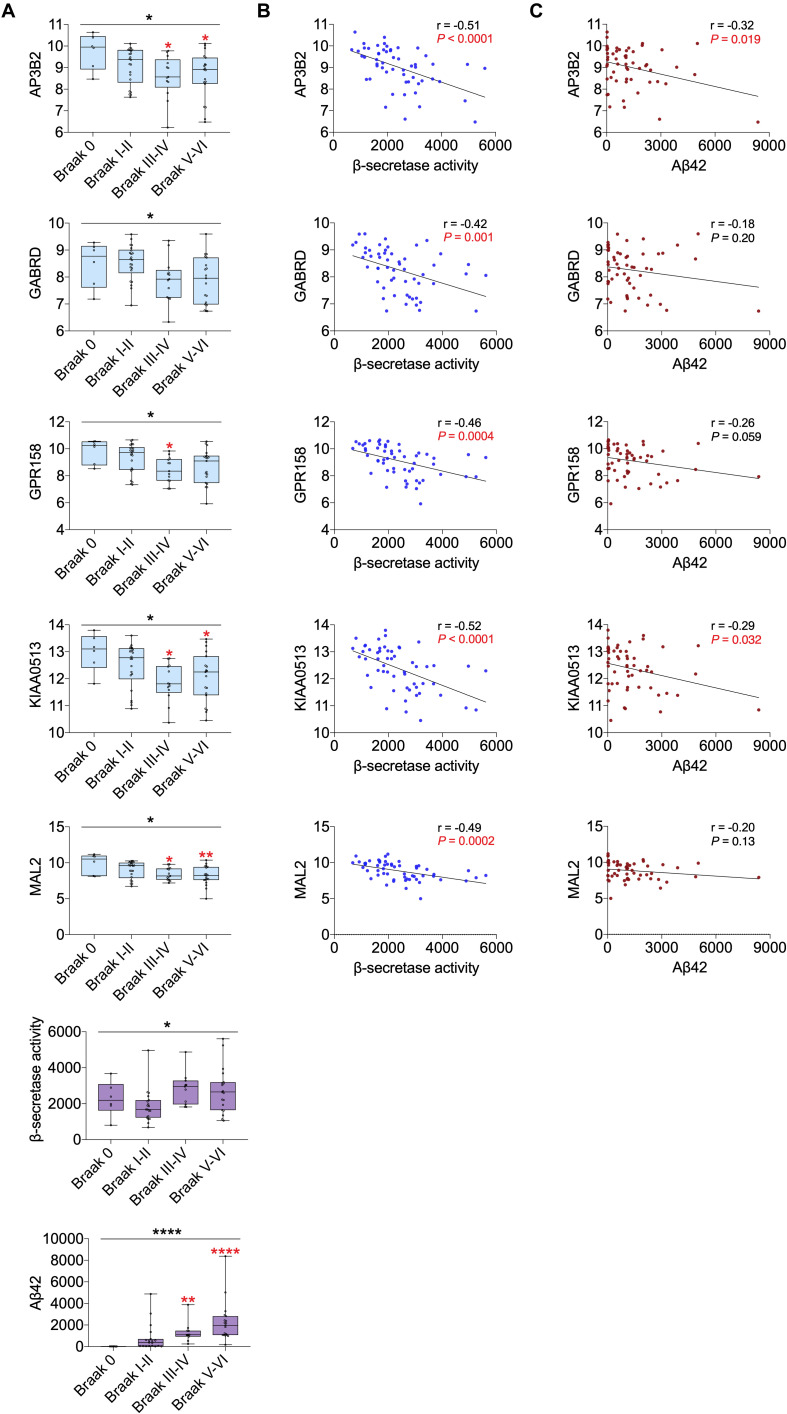
Correlation of five hub genes with β-secretase activity and Aβ42 levels using GSE106241. **(A)** The five hub gene expression, β-secretase activity, and Aβ42 levels in different Braak stages. Red asterisks indicate significant vs. Braak 0 groups. **(B)** Correlation between five hub genes and β-secretase activity. **(C)** Correlation between five hub genes and Aβ42 levels. For Panels B and C, *P* values in red are significant (*P* < 0.05). *P < 0.05; **P < 0.01; ****P < 0.0001. AD, Alzheimer disease; CN, cognitively normal.

**FIGURE 9:**
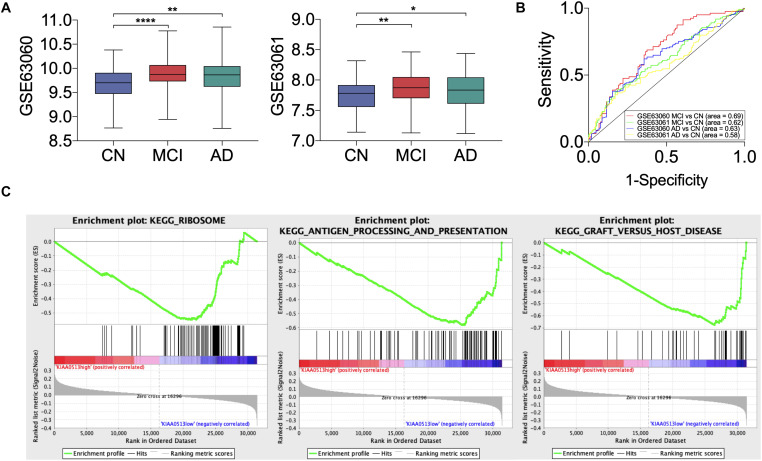
Disease-predicting ability and Gene Set Enrichment Analysis (GSEA) of *KIAA0513*. **(A)** The expression of *KIAA0513* in two blood GEO datasets, GSE63060 and GSE63061. **(B)** Receiver operating characteristic (ROC) curve of *KIAA0513* for predicting AD and MCI. **(C)** The top three GSEA terms, according to normalized enrichment scores in the low-expression group of *KIAA0513*. *P < 0.05; **P < 0.01; ****P < 0.0001. AD, Alzheimer disease; CN, cognitively normal; MCI, mild cognitive impairment.

All 19 hub genes underwent expression validation in GSE132903 (Piras et al., 2019), GSE122063 (McKay et al., 2019), GSE36980 (Hokama et al., 2014), GSE5281 (Liang et al., 2007, 2008a,b; Readhead et al., 2018), and GSE118553 (Patel et al., 2019) datasets. Except for *CRYM*, *GABRD*, *PHYHIP*, *SCN2B*, and *TERF2IP* in GSE36980 and *CAMKK2*, *PHYHIP*, and *GPR158* in GSE132903, all other hub genes were significantly down-regulated in AD samples from the five datasets (*P* < 0.05, 0.01, 0.001, or 0.0001; Figure 6 and Supplementary Figure 7). Among the 19 hub genes, we selected *AP3B2*, *GABRD*, *GPR158*, *KIAA0513*, and *MAL2*, which have been little studied on the associations with AD, in order to validate their expressions in cortices of 5xFAD and WT mice and in different Braak stages of AD samples (GSE106241; Marttinen et al., 2019). All five hub genes were down-regulated in cortices of 5xFAD mice compared to those of WT (Figure 7). We also found that the five hub genes were down-regulated as AD progressed, especially *AP3B2*, *KIAA0513*, and *MAL2*, which were significantly down-regulated in Braak III–IV and Braak V–VI when compared with Braak 0 (*P* < 0.05, 0.01, respectively, Figure 8A). β-Secretase activity and Aβ42 levels in GSE106241 were identified to be up-regulated during AD progression (Figure 8A). We found that all five hub genes were negatively associated with β-secretase activity (*P* < 0.0001, *P* = 0.001, *P* = 0.0004, *P* < 0.0001, and *P* = 0.0002, respectively, Figure 8B) and that *AP3B2* and *KIAA0513* were negatively associated with Aβ42 levels (*P* = 0.019, 0.032, respectively, Figure 8C). Next, we tested the differential expressions of the five hub genes in the two AD GEO datasets from the blood (data not shown; Sood et al., 2015) and found that *KIAA0513* was significantly up-regulated in MCI and AD samples when compared with CN samples (*P* < 0.05, 0.01, 0.0001, respectively, Figure 9A). We also found that *KIAA0513* had the ability to differentiate MCI and AD from CN in the two datasets (Figure 9B). In GSE63060, the area under the curve (AUC) for differentiating MCI and CN samples is 0.69 [95% confidence interval (CI) = 0.61–0.77], and AUC for AD and CN samples is 0.63 (95% CI = 0.56–0.70). In GSE63061, AUC for MCI and CN is 0.62 (95% CI = 0.55–0.69), and AUC for AD and CN is 0.58 (95% CI = 0.51–0.65). Furthermore, *KIAA0513* was found to be enriched in neurons of healthy human brains, as determined using an online database AlzData (Xu et al., 2018; Supplementary Figure 8)^4^.

### GSEA Reveals Potential Biological Functions of Hub Genes

We conducted GSEA of the five little-studied hub genes in the expression data of GSE118553. AD samples in GSE118553 were divided into a “high-expression group” and a “low-expression group.” We acquired 17, 9, 1, 7, and 33 significant gene sets enriched in the expression groups of *AP3B2*, *GABRD*, *GPR158*, *KIAA0513*, and *MAL2*, respectively (Supplementary Table 4). According to normalized enrichment scores, genes in the low-expressed *KIAA0513* group were mostly related to ribosome function, antigen processing and presentation, and graft-vs.-host disease (Figure 9C).

## Discussion

The current study incorporated gene expression data extracted from the temporal cortex in three GEO datasets for combined analysis and identified a key module and hub genes associated with AD via WGCNA. We believe that this is the first study to integrate combined analysis and WGCNA to identify potential hub genes as candidate biomarkers or therapeutic targets for AD using temporal cortex datasets. According to GO and KEGG analyses, the DEGs identified by combined analysis were mostly enriched in synapse function, and genes from the key module were mostly related to learning and memory, which are closely correlated with AD onset. Among these DEGs, we identified some robust genes that played vital roles in AD pathogenesis, such as *SERPINA3* (Kamboh et al., 2006), *CD163* (Pey et al., 2014), and *SST* (Duron et al., 2018; Solarski et al., 2018).

We also found 19 down-regulated hub genes. Some of these genes and their encoded proteins have been implicated in AD proceeding. For example, it was reported that aberrant Ca^2+^/calmodulin-dependent protein kinase kinase 2 (CaMKK2) may lead to the interference of iron homeostasis in the brain of AD. Also, a loss of calsyntenin-1 (CLSTN1) induced alterations to amyloid precursor protein (APP) processing (Vagnoni et al., 2012), and neuronal pentraxin 1 (NPTX1) was implicated in synaptic function dysregulation during AD progression (Cummings et al., 2017). Among these 19 hub genes, we selected five that have been little studied in AD, namely, *AP3B2*, *GABRD*, *GPR158*, *KIAA0513*, and *MAL2*, in order to explore their potential functions. Results of qRT-PCR revealed their down-regulation in 8-month 5xFAD mice, and reference mining suggested that these genes are involved in synaptic functions. *AP3B2* encodes adaptor-related protein complex 3 subunit β2 (AP3B2) that composes the neuronal isoform of adaptor-related protein complex 3 (AP-3 complex), which is involved in the sorting of synaptic vesicle proteins (Newell-Litwa et al., 2009). It was reported that *AP3B2*-knockout mouse exhibited neurobehavioral abnormalities (Anazi et al., 2017). *GABRD* encodes the delta subunit of γ-aminobutyric acid type A (GABA-A) receptor, named GABRD, which was necessary for synaptic plasticity in the hippocampi of mouse models (Whissell et al., 2016). *GPR158* encodes G protein–coupled receptor 158 (GPR158), which was found to be important in synaptic modulation, especially in the hippocampus (Khrimian et al., 2017; Condomitti et al., 2018; Sutton et al., 2018; Cetereisi et al., 2019). *KIAA0513* encodes the protein KIAA0513, which was assumed to participate in neuroplasticity and apoptosis (Lauriat et al., 2006). *MAL2* encodes a multispan transmembrane protein belonging to the myelin and lymphocyte (MAL) proteolipid family named MAL2, which was shown to be necessary as a membrane constituent of synaptic vesicles (Gronborg et al., 2010). We determined that all five hub genes were not only significantly down-regulated as AD progressed, but also in negative correlation with β-secretase activity, indicating their strong involvement existed during AD proceeding.

We also explored the diagnostic values of these five hub genes. Intriguingly, expression of *KIAA0513* was found to have a negative correlation with Aβ42 levels in the temporal cortex of AD samples and was able to distinguish MCI and AD samples from CN samples in the blood, according to ROC curves. These findings indicate that *KIAA0513* could be an interesting target for further exploration. Further investigation of *KIAA0513* revealed that it was shown to be enriched in neurons of normal human brains, suggesting that low expression level of this gene might reflect the pathological condition of the brains. Furthermore, GSEA indicated that the low-expression group of this gene was related to immune function during AD progression. In addition, its encoded protein KIAA0513 was found to potentially interact with kidney and brain expressed protein (KIBRA) *in vitro*, a cytoplasmic phosphoprotein exerting neuroprotective effects in AD (Lauriat et al., 2006; Song et al., 2019a). KIBRA was reported to be involved in exosome secretion and synaptic plasticity (Tracy et al., 2016; Song et al., 2019b), suggesting that KIAA0513 might participate in these biological functions. Because of its involvement in immune function, exosome secretion, and synaptic plasticity, a low level of expression of KIAA0513 in neurons may cause synaptic dysfunction and neuronal apoptosis during AD progression. Based on these findings, KIAA0513 could be a promising biomarker for AD diagnosis, as well as a potential target for AD treatment.

Previous microarray analyses used for combined analysis in the present study had identified genes that were involved in cellular physiological process (Liang et al., 2008a), energy metabolism (Liang et al., 2008b), gliosis (Hokama et al., 2014), and oxidative phosphorylation (McKay et al., 2019). However, after combining the three datasets (total: 54AD, 53CN), we obtained genes that mainly participated in synaptic functions, which is consistent with the results of datasets having similar or larger sample sizes (Patel et al., 2019; Piras et al., 2019). The accordance indicates that a large sample size for analysis may bring us closer to understand the genuine pathogenesis of a disease. In addition, when the resources are limited, a valid combined analysis could be of great help to enlarge the sample size.

## Conclusion

In conclusion, after integrating combined analysis and WGCNA, we identified that *AP3B2*, *GABRD*, *GPR158*, *KIAA0513*, and *MAL2*, which have been little characterized previously to be associated with AD, are vulnerable to AD. Among them, KIAA0513 was found to be a potential biomarker for early diagnosis of AD and potentially even a therapeutic target. Further research is needed to validate further the roles of these hub genes in AD pathogenesis.

## Data Availability Statement

The datasets generated for this study can be found in the online repositories. The names of the repository/repositories and accession number(s) can be found in the article/Supplementary Material.

## Ethics Statement

The animal study was reviewed and approved by the Ethics Committee of Capital Medical University.

## Author Contributions

JJ and MZ: study design. MZ and FL: data collection and analysis. MZ and LJ: experiment conducting, literature search, and article writing. JJ, MZ, LJ, and FL: article revision. All authors contributed to the article and approved the submitted version.

## Conflict of Interest

The authors declare that the research was conducted in the absence of any commercial or financial relationships that could be construed as a potential conflict of interest.

## Footnotes

1 https://www.ncbi.nlm.nih.gov/geo/

2 https://cytoscape.org

3 https://www.graphpad.com

4 http://www.alzdata.org/single_RNAseq.php
