# Supplementary material for: Identification of KIAA0513 and Other Hub Genes Associated With Alzheimer Disease Using Weighted Gene Coexpression Network Analysis
Source: Front Genet. 2020 Aug 28;11:981. doi: 10.3389/fgene.2020.00981 (PMC7483929; doi:10.3389/fgene.2020.00981)
Supplement: Supplementary file 1 [file Data_Sheet_1.DOCX]

**Supplementary materials**

**Title:** Identification of *KIAA0513* and other hub genes associated with Alzheimer’s disease using weighted gene co-expression network analysis

**Running title:** Novel hub genes in Alzheimer’s

**Min Zhu et al**

The supplementary materials are listed as follows:

**Supplementary Figure 1.** Heatmap of all differentially expressed genes.

**Supplementary Figure 2.** Volcano plot of all differentially expressed genes.

**Supplementary Figure 3.** Gene Ontology (GO) and Kyoto Encyclopedia of Genes and Genomes (KEGG) analyses of up-regulated genes in AD brains.

**Supplementary Figure 4.** Gene Ontology (GO) and Kyoto Encyclopedia of Genes and Genomes (KEGG) analyses of down-regulated genes in AD brains.

**Supplementary Figure 5.** Histogram of k and scale free topology plot.

**Supplementary Figure 6.** Scatter plots of module eigengenes in the blue, yellow and brown modules.

**Supplementary Figure 7.** Validation of expression of 14 hub genes in five datasets.

**Supplementary Figure 8.** Expression of *KIAA0513* in different cell types of healthy human brains.

**Supplementary Table 1.** Sequences of primers used in the present study.

**Supplementary Table 2.** 850 differentially expressed genes in the combined analysis.

**Supplementary Table 3.** Genes in each module.

**Supplementary Table 4.** GSEA terms of five hub genes.


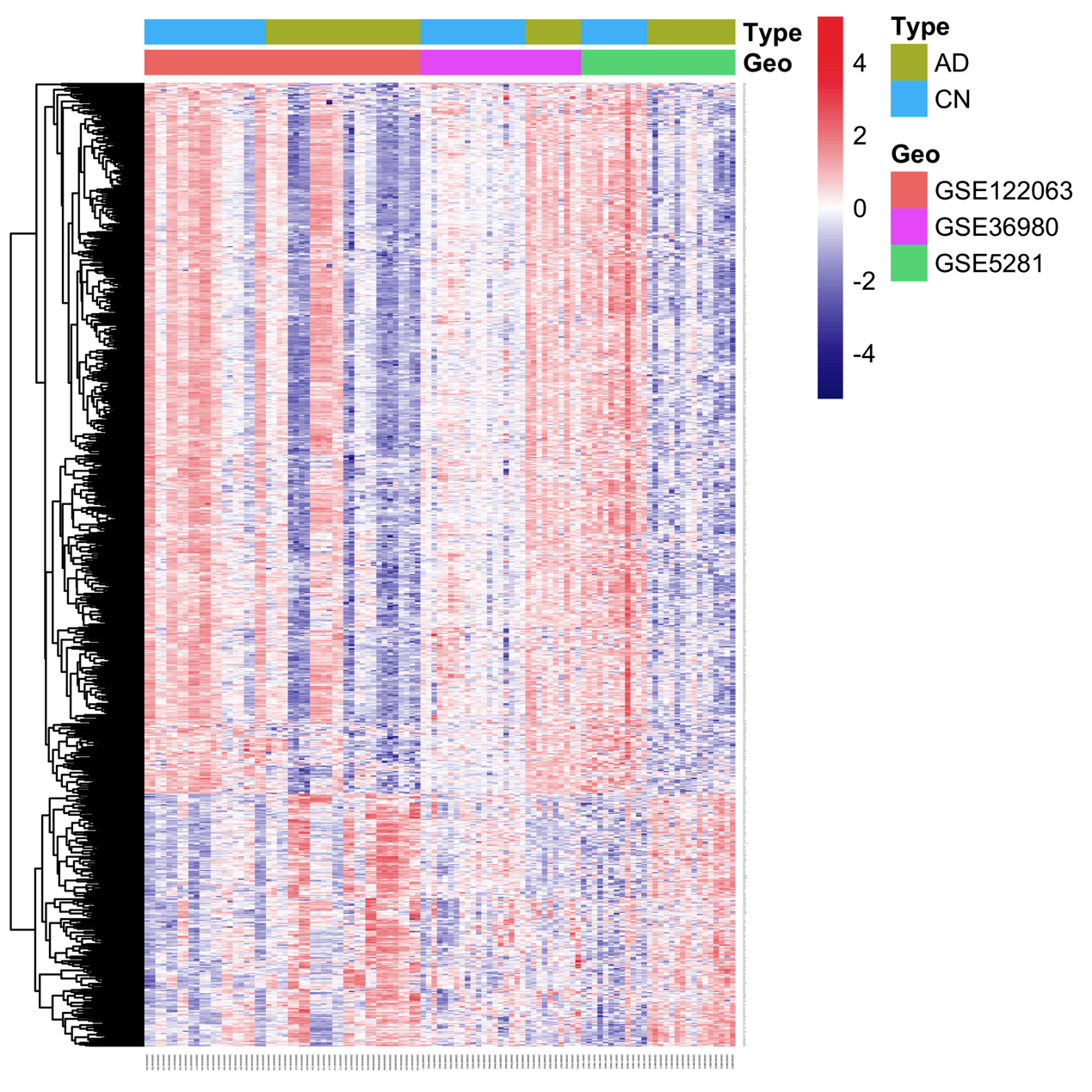


**Supplementary Figure 1. Heatmap of all differentially expressed genes.** Note: AD, Alzheimer’s disease; CN, cognitively normal; GEO, Gene Expression Omnibus; GSE, Gene Expression Omnibus Series.

**
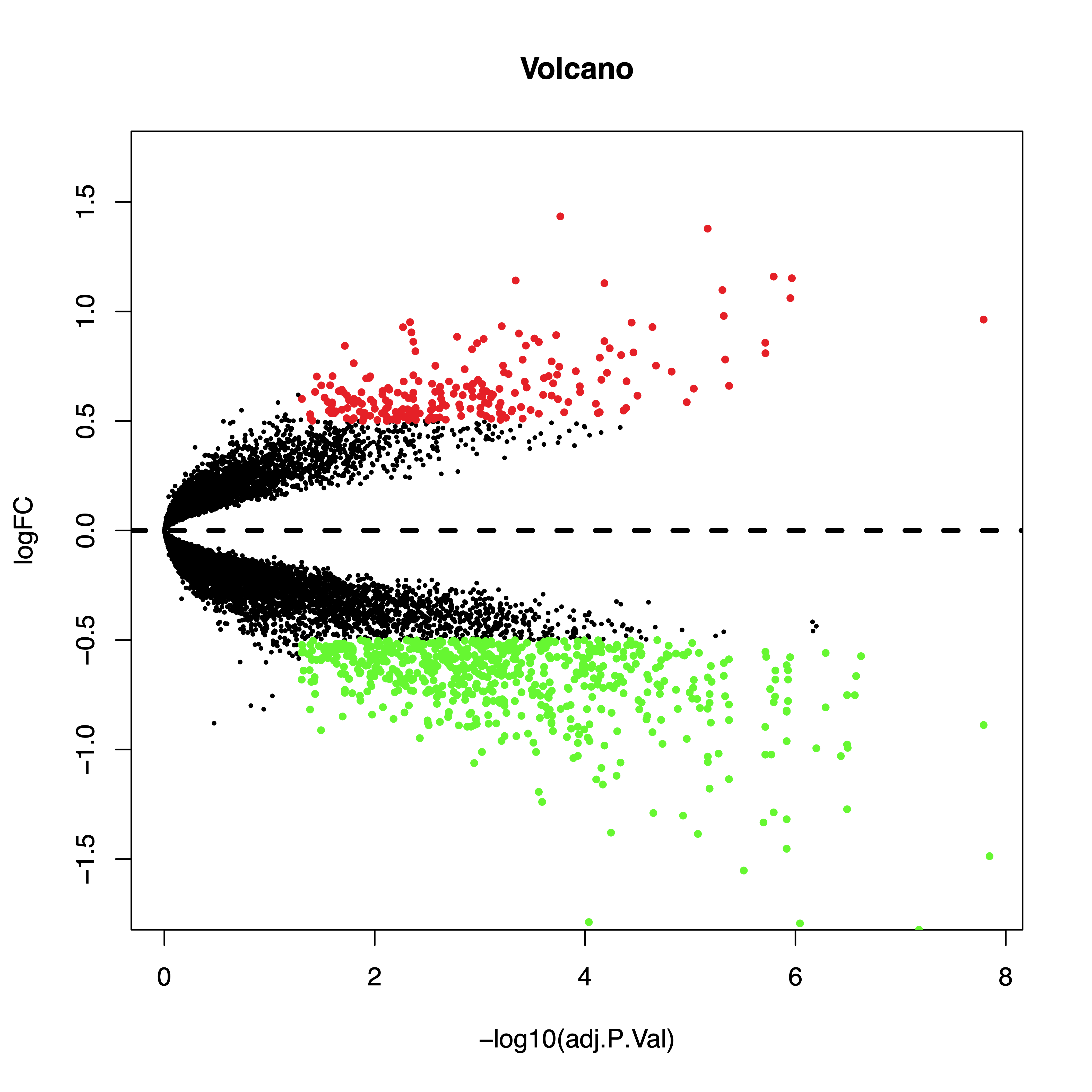
**

**Supplementary Figure 2. Volcano plot of all differentially expressed genes.** Note: FC, Fold Change.

**
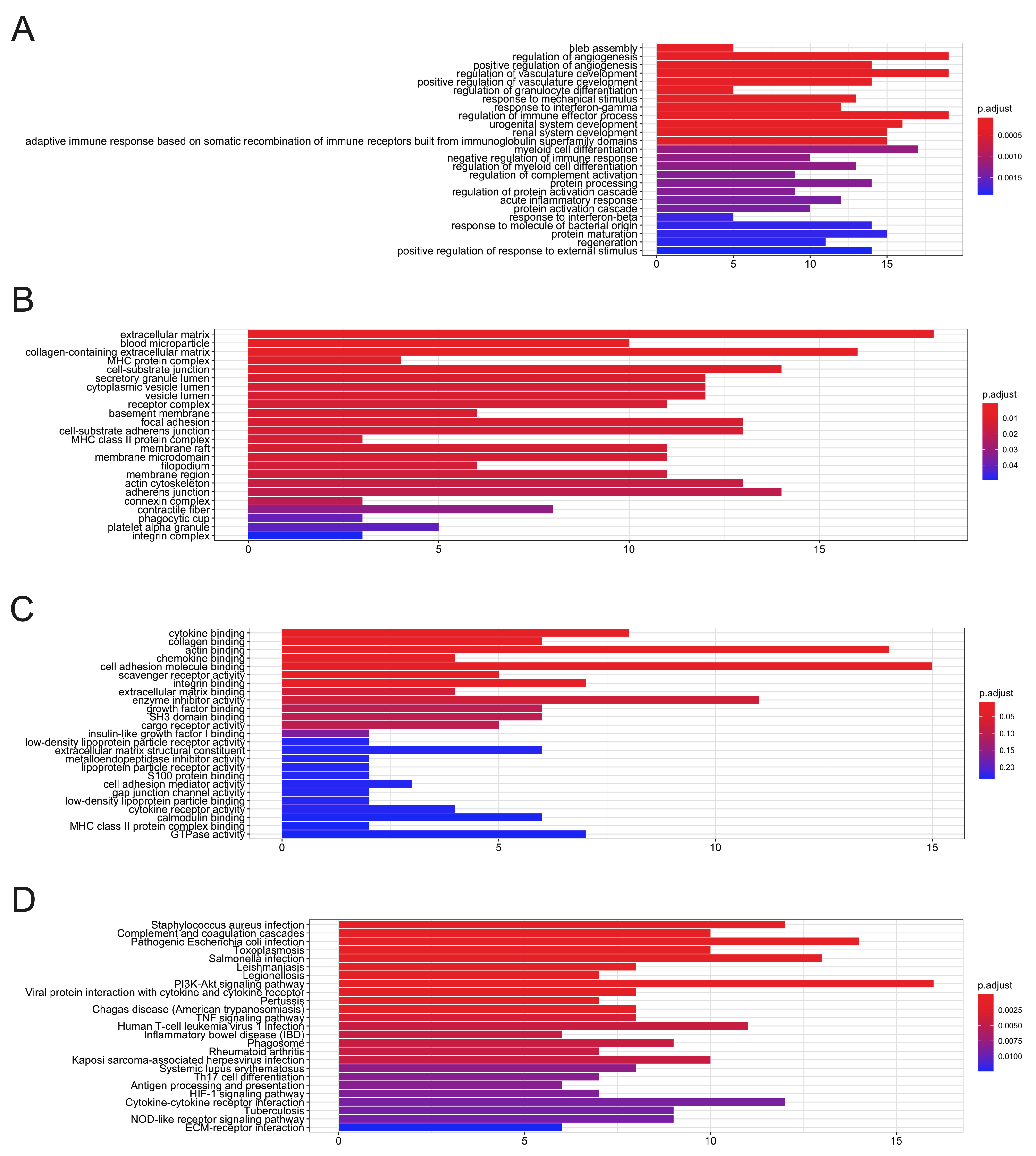
**

**Supplementary Figure 3. Gene Ontology (GO) and Kyoto Encyclopedia of Genes and Genomes (KEGG) analyses of up-regulated genes in AD brains.** (**A**) Biological processes of GO. (**B**) Cellular components of GO. (**C**) Molecular functions of GO. (**D**) KEGG pathways.


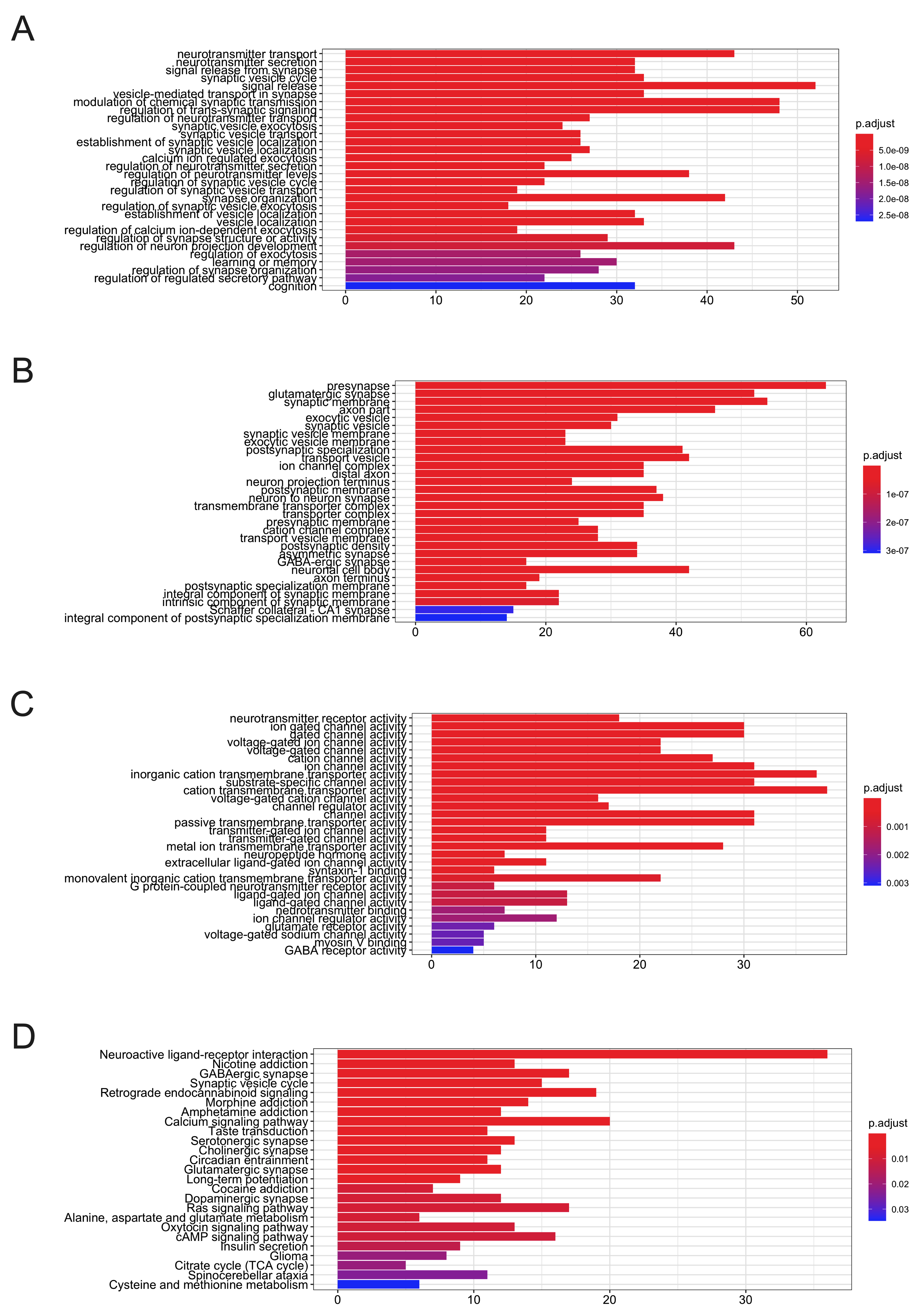


**Supplementary Figure 4. Gene Ontology (GO) and Kyoto Encyclopedia of Genes and Genomes (KEGG) analyses of down-regulated genes in AD brains.** (**A**) Biological processes of GO. (**B**) Cellular components of GO. (**C**) Molecular functions of GO. (**D**) KEGG pathways.


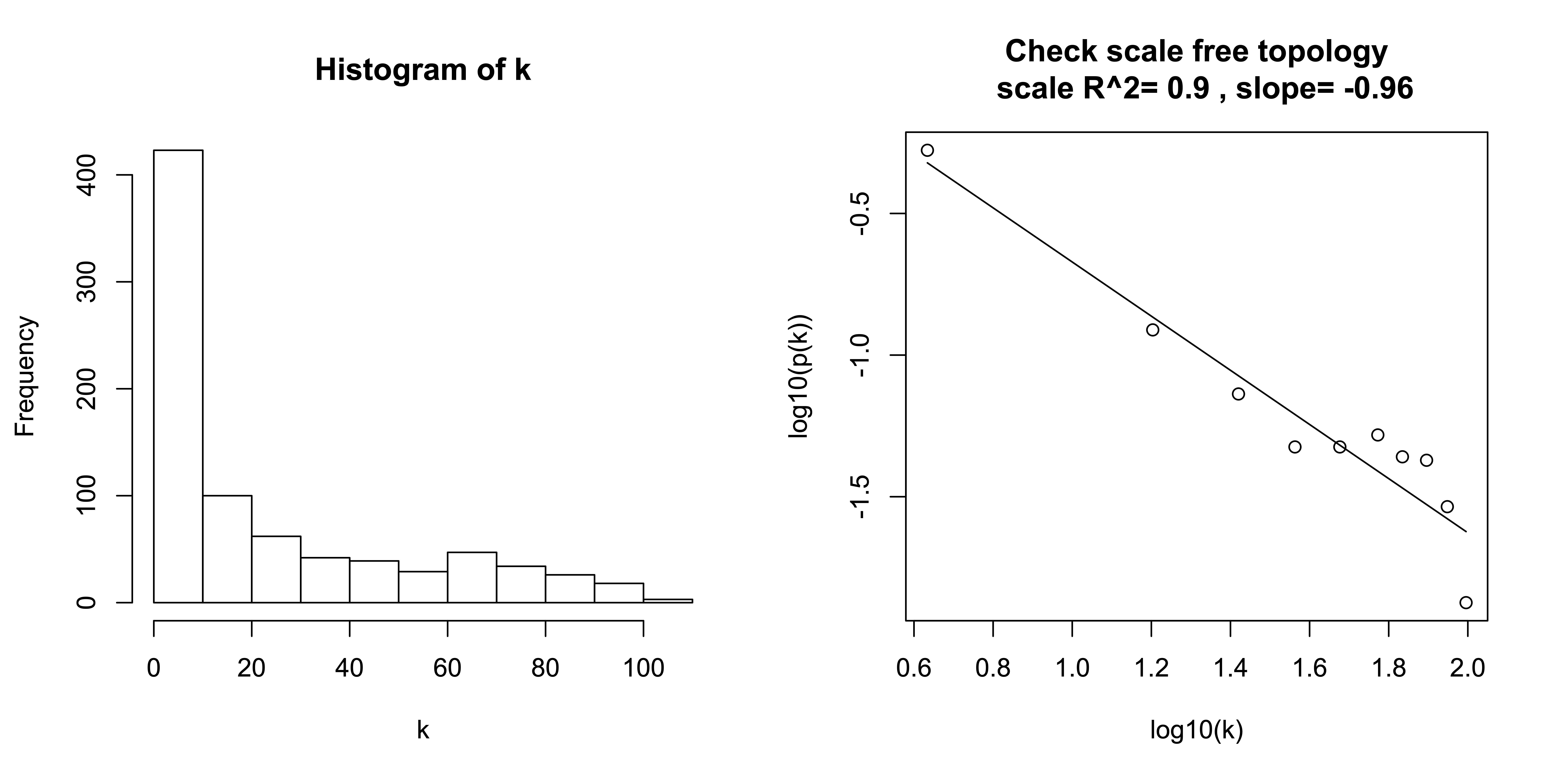


**Supplementary Figure 5. Histogram of k and scale free topology plot.** Histogram and topology plot were shown when the soft-threshold power was 5.


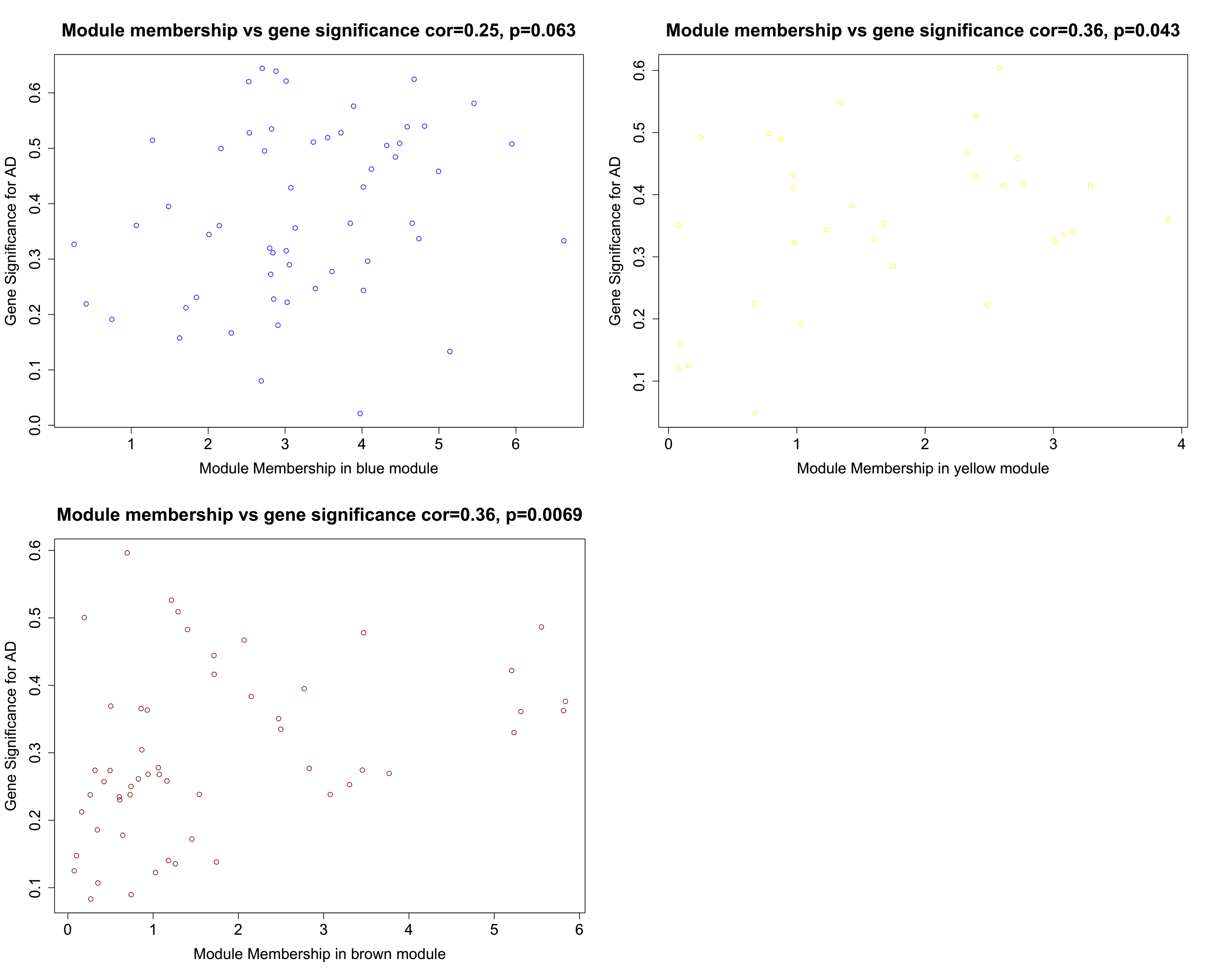


**Supplementary Figure 6. Scatter plots of module eigengenes in the blue, yellow and brown modules.**

**
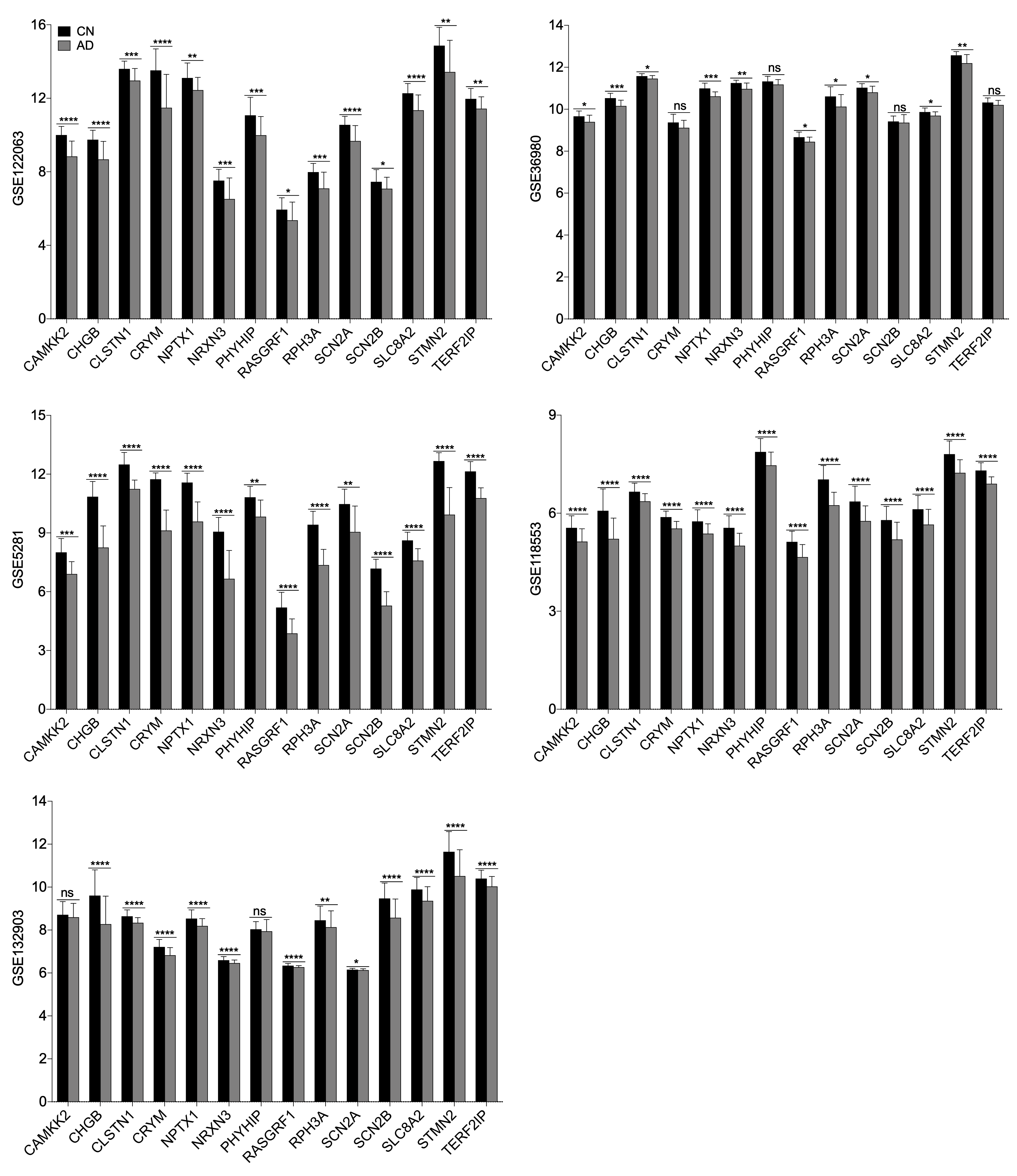
**

**Supplementary Figure 7. Expression of 14 hub genes in five datasets.** Note: *, *P* < 0.05; **, *P* < 0.01; ***, *P* < 0.001; ****, *P* < 0.0001; AD, Alzheimer’s disease; CN, Cognitively Normal; ns, no significance.


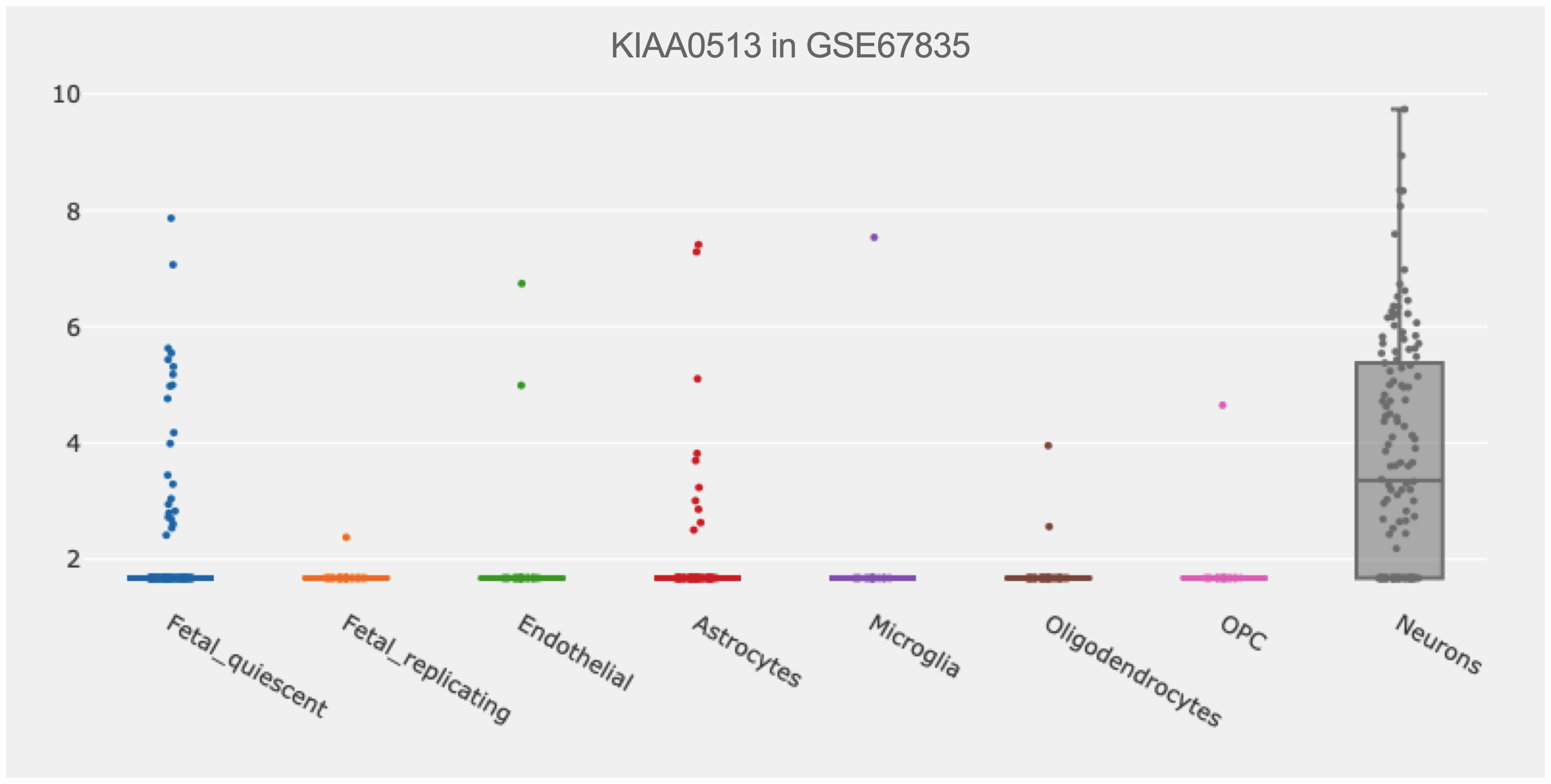


**Supplementary Figure 8. Expression of *KIAA0513* in different cell types of healthy human brains.** The figure was obtained from an online database called AlzData (<http://www.alzdata.org/single_RNAseq.php>). Note: OPC, Oligodendrocyte Precursor Cell.

**Supplementary Table 1. Sequences of primers used in the present study.**

| **Primer** | **Sequences (5'to3')** |
| --- | --- |
| mKIAA0513-F | CCAGGAGACGAGCACTAGAGGA |
| mKIAA0513-R | CTTGGAAGGTGGGAGCAGCAA |
| mAP3B2-F | CGTCAGAGTCGGAGGAGGAACA |
| mAP3B2-R | GAGGAGACACAGGCTGGACACT |
| mGABRD-F | TCACCAGTTACCGCTTCACCAC |
| mGABRD-R | ATGGCAACCAGGAGGACAGAGG |
| mGPR158-F | CCCTGCTTGATGTGTGGCTCAC |
| mGPR158-R | GCTGATTTGTGGCGGTGGAGAG |
| mMAL2-F | TCGCAGGCTTGGCAACAGAT |
| mMAL2-R | GGCACTCTTAGGTGATGGGTCA |
| mβ-actin-F | CTGTCCACCTTCCAGCAGATGT |
| mβ-actin-R | GCTCAGTAACAGTCCGCCTAGA |

Note: F, forward sequence; m, mouse; R, reverse sequence.

**Supplementary Table 2. 850 differentially expressed genes in the combined analysis.**

| **Gene** | **log Fold Change** | **P value** | **FDR** |
| --- | --- | --- | --- |
| NPTX2 | -1.486691121 | 9.09E-13 | 1.43E-08 |
| ICA1 | -0.887973179 | 2.88E-12 | 1.63E-08 |
| BACE2 | 0.963011246 | 3.10E-12 | 1.63E-08 |
| CARTPT | -1.822466528 | 1.70E-11 | 6.69E-08 |
| ASTN1 | -0.573708396 | 7.57E-11 | 2.38E-07 |
| COG1 | -0.664478892 | 1.01E-10 | 2.65E-07 |
| HPCA | -0.751742463 | 1.22E-10 | 2.73E-07 |
| FAM19A1 | -0.991944095 | 1.62E-10 | 3.19E-07 |
| MET | -1.272486158 | 2.24E-10 | 3.23E-07 |
| RPH3A | -0.976806526 | 2.19E-10 | 3.23E-07 |
| CLIP3 | -0.751484976 | 2.26E-10 | 3.23E-07 |
| TARBP1 | -1.029808967 | 2.83E-10 | 3.71E-07 |
| CAMKK2 | -0.8071908 | 4.60E-10 | 5.16E-07 |
| GSS | -0.558966392 | 4.54E-10 | 5.16E-07 |
| RGS4 | -0.994070857 | 6.45E-10 | 6.33E-07 |
| SST | -1.793612878 | 1.10E-09 | 9.05E-07 |
| SCIN | 1.151845734 | 1.38E-09 | 1.08E-06 |
| LETMD1 | -0.578421593 | 1.57E-09 | 1.12E-06 |
| GFAP | 1.061105386 | 1.49E-09 | 1.12E-06 |
| PCYOX1L | -0.778419172 | 1.72E-09 | 1.18E-06 |
| NIT2 | -0.680241578 | 1.87E-09 | 1.18E-06 |
| COPS7A | -0.638498408 | 1.82E-09 | 1.18E-06 |
| CRYM | -1.452718461 | 2.03E-09 | 1.21E-06 |
| MLIP | -1.318169357 | 2.12E-09 | 1.21E-06 |
| CHRM1 | -0.961712672 | 2.36E-09 | 1.21E-06 |
| NRN1 | -0.827067782 | 2.39E-09 | 1.21E-06 |
| STX1A | -0.822113367 | 2.20E-09 | 1.21E-06 |
| ENTPD6 | -0.614781956 | 2.28E-09 | 1.21E-06 |
| KIAA1644 | -0.680870402 | 3.17E-09 | 1.55E-06 |
| TRAPPC2L | -0.639233398 | 3.26E-09 | 1.55E-06 |
| KCNF1 | -0.757948634 | 3.38E-09 | 1.56E-06 |
| PTPN3 | -1.286865915 | 3.79E-09 | 1.61E-06 |
| BEND5 | -0.784085535 | 3.77E-09 | 1.61E-06 |
| MAFF | 1.159397447 | 3.76E-09 | 1.61E-06 |
| CHGB | -1.022832132 | 4.11E-09 | 1.70E-06 |
| MAGED1 | -0.723762592 | 4.32E-09 | 1.74E-06 |
| LIN7B | -0.576616596 | 4.83E-09 | 1.90E-06 |
| SCN3B | -1.02311094 | 5.39E-09 | 1.93E-06 |
| RGS7 | -0.896421633 | 5.54E-09 | 1.93E-06 |
| MTCH2 | -0.553782799 | 5.15E-09 | 1.93E-06 |
| SEPP1 | 0.809438888 | 5.16E-09 | 1.93E-06 |
| RFX4 | 0.857183572 | 5.43E-09 | 1.93E-06 |
| FREM3 | -1.332892054 | 5.90E-09 | 2.01E-06 |
| PPEF1 | -1.552213569 | 9.27E-09 | 3.10E-06 |
| CRYAB | 0.660780038 | 1.31E-08 | 4.27E-06 |
| TSPAN7 | -0.865369533 | 1.34E-08 | 4.28E-06 |
| ADCYAP1 | -1.135246054 | 1.41E-08 | 4.29E-06 |
| PPME1 | -0.794325949 | 1.42E-08 | 4.29E-06 |
| TOLLIP | -0.588066744 | 1.42E-08 | 4.29E-06 |
| RIMS1 | -0.756355213 | 1.60E-08 | 4.66E-06 |
| RHOJ | 0.780716619 | 1.57E-08 | 4.66E-06 |
| DDN | -0.663382807 | 1.75E-08 | 4.80E-06 |
| FAM49A | -0.604715501 | 1.77E-08 | 4.80E-06 |
| ANLN | 0.979874828 | 1.72E-08 | 4.80E-06 |
| SPP1 | 1.097874303 | 1.86E-08 | 4.95E-06 |
| GNG3 | -1.018436001 | 2.05E-08 | 5.38E-06 |
| MRPL15 | -0.690670788 | 2.48E-08 | 6.27E-06 |
| WNT10B | -0.87705916 | 2.60E-08 | 6.37E-06 |
| DLG3 | -0.618671987 | 2.60E-08 | 6.37E-06 |
| ATOH7 | -1.178450671 | 2.74E-08 | 6.55E-06 |
| CRMP1 | -0.786753342 | 2.78E-08 | 6.55E-06 |
| GULP1 | -0.746558828 | 2.80E-08 | 6.55E-06 |
| ZBBX | -1.057229803 | 3.04E-08 | 6.81E-06 |
| CACNG3 | -1.031830991 | 3.02E-08 | 6.81E-06 |
| FIBP | -0.670755608 | 2.96E-08 | 6.81E-06 |
| PTPRR | -0.813497546 | 3.13E-08 | 6.83E-06 |
| FCGBP | 1.378260008 | 3.13E-08 | 6.83E-06 |
| SPRYD7 | -0.8104304 | 3.76E-08 | 8.09E-06 |
| OSCP1 | -0.55871526 | 3.90E-08 | 8.27E-06 |
| CRH | -1.38499041 | 4.04E-08 | 8.46E-06 |
| THNSL1 | -0.768960821 | 4.17E-08 | 8.61E-06 |
| PSMA1 | -0.682251022 | 4.60E-08 | 9.26E-06 |
| MID1IP1 | 0.647726696 | 4.59E-08 | 9.26E-06 |
| OXCT1 | -0.717398652 | 4.72E-08 | 9.38E-06 |
| CYP1B1-AS1 | -0.767511022 | 4.92E-08 | 9.57E-06 |
| PIAS2 | -0.513515664 | 4.94E-08 | 9.57E-06 |
| PFN2 | -0.739677497 | 5.29E-08 | 1.01E-05 |
| DNAJC19 | -0.555246627 | 5.54E-08 | 1.05E-05 |
| SV2B | -0.95106977 | 5.84E-08 | 1.08E-05 |
| B2M | 0.585844764 | 5.85E-08 | 1.08E-05 |
| MDH2 | -0.570637245 | 6.21E-08 | 1.13E-05 |
| STAT4 | -1.301470102 | 6.49E-08 | 1.17E-05 |
| YWHAZ | -0.567776246 | 6.57E-08 | 1.17E-05 |
| RAB3C | -0.8145032 | 7.54E-08 | 1.31E-05 |
| SSTR1 | -0.727069846 | 8.00E-08 | 1.37E-05 |
| CACNB3 | -0.561533512 | 8.00E-08 | 1.37E-05 |
| HSPA2 | 0.725432758 | 8.91E-08 | 1.50E-05 |
| SCCPDH | -0.57167071 | 9.62E-08 | 1.60E-05 |
| TLN2 | -0.525595504 | 9.70E-08 | 1.60E-05 |
| PDHB | -0.577835992 | 1.03E-07 | 1.68E-05 |
| LRRC7 | -0.564594044 | 1.04E-07 | 1.68E-05 |
| DDX25 | -0.687095657 | 1.10E-07 | 1.76E-05 |
| SVOP | -0.974000513 | 1.16E-07 | 1.84E-05 |
| AKAP5 | -0.864256241 | 1.22E-07 | 1.89E-05 |
| ERLEC1 | -0.592209807 | 1.20E-07 | 1.89E-05 |
| SLIT1 | -0.714474339 | 1.26E-07 | 1.94E-05 |
| BSN | -0.745974552 | 1.29E-07 | 1.95E-05 |
| SLC8A2 | -0.619108197 | 1.28E-07 | 1.95E-05 |
| NNAT | -0.702189871 | 1.39E-07 | 2.06E-05 |
| PTDSS1 | -0.500223453 | 1.38E-07 | 2.06E-05 |
| EPS8 | 0.752937957 | 1.44E-07 | 2.12E-05 |
| KCTD1 | -0.627890186 | 1.46E-07 | 2.13E-05 |
| HSPB3 | -1.289640552 | 1.57E-07 | 2.24E-05 |
| HTR2A | -0.920633315 | 1.63E-07 | 2.29E-05 |
| AEBP1 | 0.928901572 | 1.63E-07 | 2.29E-05 |
| P4HTM | -0.678046889 | 1.79E-07 | 2.49E-05 |
| MTX2 | -0.738535018 | 1.87E-07 | 2.55E-05 |
| ATL1 | -0.846433243 | 1.90E-07 | 2.56E-05 |
| BCAS2 | -0.658525494 | 1.91E-07 | 2.56E-05 |
| CDK5 | -0.862287005 | 1.96E-07 | 2.61E-05 |
| ATP6V1E1 | -0.785000898 | 2.22E-07 | 2.90E-05 |
| MAPK9 | -0.561267075 | 2.24E-07 | 2.90E-05 |
| GABRB3 | -0.568738343 | 2.28E-07 | 2.93E-05 |
| MBOAT7 | -0.528041085 | 2.37E-07 | 3.00E-05 |
| SLC9A6 | -0.816635709 | 2.47E-07 | 3.05E-05 |
| RAB15 | -0.55321861 | 2.44E-07 | 3.05E-05 |
| LRRTM1 | -0.773940436 | 2.54E-07 | 3.11E-05 |
| ANP32B | 0.615444904 | 2.61E-07 | 3.18E-05 |
| XKR4 | -0.608582407 | 2.75E-07 | 3.30E-05 |
| MKKS | -0.520125248 | 2.74E-07 | 3.30E-05 |
| CYFIP2 | -0.572127641 | 2.93E-07 | 3.47E-05 |
| CXCR4 | 0.813206482 | 2.94E-07 | 3.47E-05 |
| NDUFA9 | -0.522731988 | 3.09E-07 | 3.61E-05 |
| GBP2 | 0.949212058 | 3.12E-07 | 3.61E-05 |
| VSTM2L | -0.634581595 | 3.20E-07 | 3.67E-05 |
| CHMP1B | -0.501735072 | 3.27E-07 | 3.70E-05 |
| KLHDC9 | -0.523066616 | 3.56E-07 | 3.98E-05 |
| FH | -0.61996905 | 3.62E-07 | 4.01E-05 |
| PFKFB3 | 0.681434926 | 3.67E-07 | 4.03E-05 |
| ITPKB | 0.559396525 | 3.76E-07 | 4.07E-05 |
| UBE2N | -0.742882071 | 4.03E-07 | 4.31E-05 |
| PADI2 | 0.547767883 | 4.03E-07 | 4.31E-05 |
| PDK3 | -0.554360502 | 4.07E-07 | 4.32E-05 |
| NCALD | -0.691459096 | 4.16E-07 | 4.34E-05 |
| SPINT2 | -0.57947853 | 4.17E-07 | 4.34E-05 |
| ERMN | 0.800883253 | 4.42E-07 | 4.54E-05 |
| INA | -1.059451663 | 4.55E-07 | 4.60E-05 |
| ST8SIA3 | -0.668093031 | 4.65E-07 | 4.65E-05 |
| NECAB2 | -0.916495229 | 5.00E-07 | 4.95E-05 |
| DDAH1 | -0.531402235 | 5.01E-07 | 4.95E-05 |
| ACTR10 | -0.699204108 | 5.07E-07 | 4.97E-05 |
| CLSTN1 | -0.521277356 | 5.11E-07 | 4.98E-05 |
| NEUROD6 | -1.119677256 | 5.18E-07 | 5.02E-05 |
| RWDD2A | -0.584783945 | 5.23E-07 | 5.03E-05 |
| PACSIN1 | -0.662669725 | 5.46E-07 | 5.20E-05 |
| RASL10A | -0.542491365 | 5.68E-07 | 5.34E-05 |
| LNX1 | -0.719632843 | 5.73E-07 | 5.36E-05 |
| CA10 | -0.832246887 | 6.06E-07 | 5.60E-05 |
| CALB1 | -1.379162026 | 6.17E-07 | 5.67E-05 |
| PGRMC1 | -0.70812562 | 6.43E-07 | 5.84E-05 |
| RHBDF2 | 0.832013652 | 6.40E-07 | 5.84E-05 |
| GPATCH2 | -0.539454293 | 6.50E-07 | 5.87E-05 |
| IMPAD1 | -0.535539694 | 6.60E-07 | 5.92E-05 |
| MYO10 | 0.720458569 | 6.98E-07 | 6.19E-05 |
| BDNF | -0.981875486 | 7.48E-07 | 6.55E-05 |
| NUPR1 | 0.864681712 | 7.51E-07 | 6.55E-05 |
| APLNR | 1.129236102 | 7.47E-07 | 6.55E-05 |
| CISD1 | -0.718482925 | 7.82E-07 | 6.71E-05 |
| RUNDC1 | -0.618730068 | 7.78E-07 | 6.71E-05 |
| STMN2 | -1.159631461 | 7.94E-07 | 6.78E-05 |
| TAGLN3 | -1.083821996 | 8.47E-07 | 7.00E-05 |
| ATRNL1 | -0.817317288 | 8.38E-07 | 7.00E-05 |
| FBXL16 | -0.556382376 | 8.45E-07 | 7.00E-05 |
| LINGO2 | -0.525380529 | 8.30E-07 | 7.00E-05 |
| SLC4A11 | 0.688254077 | 8.40E-07 | 7.00E-05 |
| RBP4 | -0.814057413 | 8.60E-07 | 7.04E-05 |
| COX18 | -0.659414857 | 8.74E-07 | 7.11E-05 |
| GEM | 0.789216157 | 8.95E-07 | 7.25E-05 |
| DNM1L | -0.516185452 | 9.03E-07 | 7.26E-05 |
| ZNF652 | 0.540208781 | 9.06E-07 | 7.26E-05 |
| SEZ6L | -0.631691676 | 9.22E-07 | 7.35E-05 |
| PPP4R4 | -0.533943925 | 9.39E-07 | 7.45E-05 |
| DDX28 | -0.501045802 | 9.51E-07 | 7.50E-05 |
| DCLK1 | -0.683097829 | 9.68E-07 | 7.56E-05 |
| PREX1 | 0.535923937 | 9.63E-07 | 7.56E-05 |
| TMEM59L | -0.703447695 | 9.78E-07 | 7.60E-05 |
| ENC1 | -1.13631367 | 1.01E-06 | 7.83E-05 |
| DTX3L | 0.579019913 | 1.03E-06 | 7.90E-05 |
| ATP8A2 | -0.552431369 | 1.04E-06 | 7.93E-05 |
| C14orf79 | -0.674455372 | 1.09E-06 | 8.26E-05 |
| ATP6V1C1 | -0.540318036 | 1.11E-06 | 8.37E-05 |
| OPN3 | -0.759772639 | 1.14E-06 | 8.53E-05 |
| SPOCK1 | -0.614090886 | 1.14E-06 | 8.53E-05 |
| NPTX1 | -0.713139238 | 1.18E-06 | 8.73E-05 |
| FKBP1B | -0.627272992 | 1.18E-06 | 8.73E-05 |
| RASAL1 | -0.554288118 | 1.20E-06 | 8.77E-05 |
| MLLT11 | -0.961138605 | 1.26E-06 | 9.07E-05 |
| C3orf14 | -0.572478819 | 1.26E-06 | 9.07E-05 |
| DIRAS1 | -0.550305243 | 1.25E-06 | 9.07E-05 |
| PPIA | -0.519813277 | 1.25E-06 | 9.07E-05 |
| FRMPD4 | -0.884610178 | 1.27E-06 | 9.08E-05 |
| SPATA7 | -0.549151857 | 1.30E-06 | 9.18E-05 |
| PCSK1 | -1.787784086 | 1.31E-06 | 9.21E-05 |
| YWHAB | -0.581297539 | 1.35E-06 | 9.40E-05 |
| CCT6B | -0.945311804 | 1.37E-06 | 9.43E-05 |
| LOC151760 | -0.815597015 | 1.41E-06 | 9.59E-05 |
| DIEXF | -0.58427265 | 1.40E-06 | 9.59E-05 |
| FXYD7 | -0.620234734 | 1.49E-06 | 0.000100087 |
| ATP5B | -0.653694867 | 1.50E-06 | 0.000100408 |
| SYN2 | -0.71003852 | 1.53E-06 | 0.000101642 |
| CAMK2D | -0.501142102 | 1.53E-06 | 0.000101642 |
| SULT4A1 | -0.908042597 | 1.54E-06 | 0.000101684 |
| IDH3G | -0.634833541 | 1.56E-06 | 0.000101922 |
| ARHGDIG | -0.609568015 | 1.61E-06 | 0.000104209 |
| NUDT7 | -0.580473011 | 1.62E-06 | 0.000104863 |
| TTLL1 | -0.609961906 | 1.63E-06 | 0.00010487 |
| FBXO4 | -0.625826583 | 1.64E-06 | 0.000105316 |
| CHRM3 | -0.649991564 | 1.71E-06 | 0.000109304 |
| ANKRD16 | -0.653412978 | 1.74E-06 | 0.000110517 |
| BOK | 0.632106484 | 1.76E-06 | 0.000111397 |
| TNFRSF1A | 0.658614195 | 1.79E-06 | 0.000112221 |
| NRSN1 | -0.931033688 | 1.83E-06 | 0.000114293 |
| CBLN4 | -1.02891813 | 1.90E-06 | 0.000117222 |
| SLC22A6 | -0.969944658 | 1.90E-06 | 0.000117222 |
| ATCAY | -0.505870713 | 1.89E-06 | 0.000117222 |
| ANO3 | -0.895798301 | 1.91E-06 | 0.000117453 |
| CHCHD6 | -0.661830371 | 1.93E-06 | 0.000117809 |
| SNX4 | -0.528754459 | 1.95E-06 | 0.000118815 |
| LINGO1 | -0.568100787 | 1.97E-06 | 0.000119568 |
| STMN1 | -0.647406121 | 2.01E-06 | 0.00012065 |
| BST2 | 0.727265715 | 2.06E-06 | 0.000122496 |
| ATP5A1 | -0.679319923 | 2.14E-06 | 0.000126165 |
| SYP | -1.038875777 | 2.24E-06 | 0.000129161 |
| ZCCHC12 | -0.904296953 | 2.38E-06 | 0.000135772 |
| EPHA5 | -0.861510831 | 2.43E-06 | 0.000137365 |
| SNCA | -0.796579548 | 2.43E-06 | 0.000137365 |
| B9D1 | -0.593347486 | 2.55E-06 | 0.000142398 |
| WDR86 | -0.723168449 | 2.58E-06 | 0.000143381 |
| UNC5B | 0.586478251 | 2.60E-06 | 0.000144124 |
| HAPLN1 | -0.717050659 | 2.65E-06 | 0.000146321 |
| TXNDC9 | -0.532773457 | 2.69E-06 | 0.000147551 |
| RAB27B | -0.942727451 | 2.73E-06 | 0.000148875 |
| RNF175 | -0.80256417 | 2.73E-06 | 0.000148875 |
| APOO | -0.616353311 | 2.85E-06 | 0.000154809 |
| JAKMIP1 | -0.575893692 | 2.90E-06 | 0.000157139 |
| CLMN | 0.539699803 | 2.93E-06 | 0.000158028 |
| KCNQ3 | -0.50521585 | 2.94E-06 | 0.000158118 |
| GOT1 | -0.814602202 | 3.20E-06 | 0.000170738 |
| SERPINA3 | 1.434273525 | 3.23E-06 | 0.000172014 |
| IFI16 | 0.748027512 | 3.33E-06 | 0.000176037 |
| PECAM1 | 0.59992003 | 3.45E-06 | 0.00018113 |
| FAM102B | -0.577625387 | 3.56E-06 | 0.000184386 |
| CCR1 | 0.711639639 | 3.55E-06 | 0.000184386 |
| C1QC | 0.8919688 | 3.66E-06 | 0.000188257 |
| TERF2IP | -0.509036644 | 3.76E-06 | 0.000192778 |
| LPAR1 | 0.671999058 | 3.93E-06 | 0.000201036 |
| PPP1R14C | -0.915277984 | 3.96E-06 | 0.000202064 |
| GAD1 | -0.84447358 | 4.07E-06 | 0.000205651 |
| CAMK4 | -0.73138454 | 4.12E-06 | 0.000207473 |
| CLIC1 | 0.616195125 | 4.16E-06 | 0.000208003 |
| GMPR | 0.772123902 | 4.16E-06 | 0.000208003 |
| CAMK1G | -0.881382127 | 4.21E-06 | 0.000208623 |
| REEP1 | -0.875300172 | 4.21E-06 | 0.000208623 |
| GPR88 | -0.584139471 | 4.34E-06 | 0.000212949 |
| LHFPL4 | -0.503974979 | 4.34E-06 | 0.000212949 |
| RAB3A | -0.580739854 | 4.41E-06 | 0.000215643 |
| FAR2 | -0.818251795 | 4.46E-06 | 0.000216845 |
| GLMN | -0.692942675 | 4.45E-06 | 0.000216845 |
| DGKI | -0.741748698 | 4.51E-06 | 0.000217947 |
| IGF1 | -0.572375347 | 4.57E-06 | 0.00021997 |
| RGS17 | -0.763683612 | 4.62E-06 | 0.0002217 |
| CDH10 | -0.513787241 | 4.65E-06 | 0.000222161 |
| FAM107B | 0.705194322 | 4.65E-06 | 0.000222161 |
| RWDD2B | -0.817990063 | 4.75E-06 | 0.000224661 |
| SLC9A7 | -0.502178714 | 4.76E-06 | 0.000224662 |
| MIR7-3HG | -0.756655541 | 4.84E-06 | 0.000226681 |
| CITED1 | -0.84257163 | 4.89E-06 | 0.000228717 |
| ZWILCH | -0.803848915 | 5.17E-06 | 0.000236722 |
| TMX4 | -0.535697315 | 5.16E-06 | 0.000236722 |
| PKNOX2 | -0.518617563 | 5.12E-06 | 0.000236722 |
| RAB3B | -0.622995283 | 5.42E-06 | 0.000245305 |
| EHD3 | -0.507413744 | 5.41E-06 | 0.000245305 |
| THY1 | -0.651779953 | 5.49E-06 | 0.000246084 |
| TNFAIP3 | 0.695734904 | 5.48E-06 | 0.000246084 |
| KIF1C | 0.619402905 | 5.61E-06 | 0.000250177 |
| STEAP2 | -0.710793356 | 5.65E-06 | 0.000250642 |
| VSNL1 | -1.238829271 | 5.79E-06 | 0.000255948 |
| MEST | -0.537072516 | 5.84E-06 | 0.000257765 |
| GABRA5 | -0.795841753 | 5.94E-06 | 0.000261317 |
| PI4KA | -0.549562235 | 6.01E-06 | 0.000262884 |
| GABBR2 | -0.753977905 | 6.13E-06 | 0.000266591 |
| NRXN3 | -0.855806292 | 6.21E-06 | 0.000269401 |
| GRIN2B | -0.522961557 | 6.33E-06 | 0.000272919 |
| QKI | 0.533529603 | 6.44E-06 | 0.000275315 |
| ADAMTS1 | 0.860413512 | 6.41E-06 | 0.000275315 |
| MAL2 | -1.193055902 | 6.48E-06 | 0.000275749 |
| KCNS2 | -0.740714253 | 6.69E-06 | 0.000283788 |
| NELL2 | -1.011093693 | 6.90E-06 | 0.000292221 |
| PDE1A | -0.703703989 | 7.10E-06 | 0.000298169 |
| BCAS1 | 0.876827408 | 7.29E-06 | 0.000303431 |
| KCNV1 | -0.968620374 | 7.48E-06 | 0.000309858 |
| GPRASP1 | -0.751822053 | 7.65E-06 | 0.000315143 |
| KIAA0513 | -0.577906497 | 7.79E-06 | 0.000320234 |
| FGF12 | -0.727266882 | 7.83E-06 | 0.000321046 |
| NDRG4 | -0.646116023 | 7.95E-06 | 0.000324439 |
| C1R | 0.550377296 | 8.14E-06 | 0.000329325 |
| SLC25A14 | -0.605611422 | 8.25E-06 | 0.000332976 |
| HS6ST3 | -0.580238841 | 8.63E-06 | 0.000341189 |
| TMEM132D | -0.67383275 | 8.67E-06 | 0.000342013 |
| GDA | -0.927002648 | 8.86E-06 | 0.000348583 |
| PHYHIPL | -0.51224078 | 9.07E-06 | 0.000356109 |
| CXCL16 | 0.653032813 | 9.18E-06 | 0.000359195 |
| VASP | 0.844419095 | 9.38E-06 | 0.000365344 |
| UGT8 | 0.680027379 | 9.64E-06 | 0.000374535 |
| GJC1 | 0.510841548 | 1.03E-05 | 0.000391877 |
| AZGP1 | 0.779941563 | 1.03E-05 | 0.00039208 |
| RCAN2 | -0.533244983 | 1.06E-05 | 0.000399955 |
| CHN1 | -0.845992677 | 1.07E-05 | 0.000402402 |
| PLD3 | -0.738414775 | 1.07E-05 | 0.000402402 |
| FSTL4 | -0.605176083 | 1.07E-05 | 0.000402402 |
| CMTM3 | 0.563850559 | 1.09E-05 | 0.000406409 |
| WDR54 | -0.655589099 | 1.12E-05 | 0.000415175 |
| EVI2B | 0.899456434 | 1.16E-05 | 0.000425534 |
| TASP1 | -0.642414345 | 1.18E-05 | 0.000432623 |
| CCDC113 | -0.626759272 | 1.19E-05 | 0.000435555 |
| GNPDA2 | -0.552959849 | 1.20E-05 | 0.000439116 |
| GOLT1A | -0.938793079 | 1.24E-05 | 0.000449942 |
| ZNF215 | -0.692776188 | 1.25E-05 | 0.000449942 |
| FBXW7 | -0.540268902 | 1.25E-05 | 0.000449942 |
| GOT2 | -0.588317299 | 1.28E-05 | 0.000456908 |
| CD163 | 1.141589713 | 1.27E-05 | 0.000456908 |
| ADAM23 | -0.545341485 | 1.29E-05 | 0.000459994 |
| PARP2 | -0.500548066 | 1.30E-05 | 0.000463625 |
| SLA | 0.628247605 | 1.31E-05 | 0.000464423 |
| GAP43 | -0.778779867 | 1.32E-05 | 0.00046532 |
| MECR | -0.569724883 | 1.32E-05 | 0.00046532 |
| DNAH1 | -0.518389082 | 1.36E-05 | 0.000479328 |
| STYK1 | -0.661279909 | 1.38E-05 | 0.000480566 |
| CAMKV | -0.585653292 | 1.37E-05 | 0.000480566 |
| INPP5D | 0.551261539 | 1.42E-05 | 0.000492453 |
| GOLIM4 | 0.543478278 | 1.45E-05 | 0.000500902 |
| STXBP5L | -0.52244575 | 1.51E-05 | 0.000519952 |
| NRGN | -0.665526783 | 1.55E-05 | 0.000528705 |
| CCNH | -0.511593853 | 1.55E-05 | 0.000528705 |
| GARS | -0.548210375 | 1.56E-05 | 0.000532127 |
| EMP1 | 0.714198667 | 1.58E-05 | 0.000534275 |
| DLGAP2 | -0.832573907 | 1.68E-05 | 0.000559491 |
| PTK2B | -0.543259727 | 1.67E-05 | 0.000559491 |
| SEZ6L2 | -0.605776725 | 1.69E-05 | 0.000561177 |
| AKR1C3 | 0.514964813 | 1.73E-05 | 0.000565535 |
| CD200 | -0.93833746 | 1.79E-05 | 0.000582317 |
| TUBA4A | -0.705383622 | 1.79E-05 | 0.000582317 |
| LMBRD2 | -0.525538774 | 1.79E-05 | 0.000582317 |
| HPRT1 | -0.800309818 | 1.80E-05 | 0.000582759 |
| C2orf80 | -0.77938268 | 1.81E-05 | 0.000585945 |
| SRGN | 0.721470541 | 1.83E-05 | 0.000589378 |
| SYN1 | -0.628309892 | 1.85E-05 | 0.000593698 |
| GPHN | -0.536511844 | 1.85E-05 | 0.000593698 |
| NAPB | -0.648316743 | 1.89E-05 | 0.000602546 |
| SLC7A7 | 0.75317586 | 1.88E-05 | 0.000602546 |
| PRKCG | -0.565425872 | 1.92E-05 | 0.000606657 |
| BCL6 | 0.528020321 | 1.92E-05 | 0.000606657 |
| NGEF | -0.651480412 | 1.93E-05 | 0.000607026 |
| VTA1 | -0.540550476 | 1.93E-05 | 0.000607026 |
| COL24A1 | -0.770192495 | 1.94E-05 | 0.000611216 |
| VSIG4 | 0.932876878 | 1.98E-05 | 0.000620186 |
| GPR158 | -0.960571782 | 2.00E-05 | 0.000622902 |
| BASP1 | -0.662839998 | 2.00E-05 | 0.000622902 |
| PART1 | -0.620523715 | 2.08E-05 | 0.00063594 |
| CNTNAP5 | -0.54080422 | 2.07E-05 | 0.00063594 |
| ARPC1B | 0.505344568 | 2.07E-05 | 0.00063594 |
| MT1X | 0.583151007 | 2.08E-05 | 0.00063594 |
| BFSP1 | -0.786408842 | 2.15E-05 | 0.000648378 |
| TGFBR1 | 0.535672167 | 2.15E-05 | 0.000648378 |
| ZIC2 | 0.64683 | 2.17E-05 | 0.000651374 |
| NAP1L5 | -0.773222172 | 2.18E-05 | 0.000653659 |
| SASH1 | 0.565423619 | 2.20E-05 | 0.00065814 |
| FIG4 | -0.519806503 | 2.22E-05 | 0.000662541 |
| SLC25A4 | -0.588038343 | 2.24E-05 | 0.000664657 |
| SLC25A12 | -0.654474943 | 2.30E-05 | 0.000679998 |
| CECR6 | -0.613163086 | 2.32E-05 | 0.000682076 |
| PGM2L1 | -0.650832752 | 2.33E-05 | 0.000682932 |
| HMGCS1 | -0.586254717 | 2.37E-05 | 0.000693312 |
| PNOC | -0.880497095 | 2.40E-05 | 0.000699198 |
| TUBB2A | -0.590882344 | 2.40E-05 | 0.000699198 |
| TRIM36 | -0.697134867 | 2.50E-05 | 0.000721891 |
| SCN2A | -0.641908775 | 2.59E-05 | 0.000744221 |
| MYL5 | -0.77803174 | 2.60E-05 | 0.000744779 |
| DDIT4L | 0.620678858 | 2.62E-05 | 0.000750289 |
| GNG2 | -0.735714292 | 2.66E-05 | 0.000756132 |
| FBXO34 | -0.632686338 | 2.68E-05 | 0.000760715 |
| ITFG1 | -0.52031936 | 2.72E-05 | 0.000764526 |
| UNC80 | -0.59188424 | 2.73E-05 | 0.000766974 |
| FABP3 | -0.572607157 | 2.79E-05 | 0.000778691 |
| NIPAL2 | -0.514698193 | 2.79E-05 | 0.000778691 |
| MAP2 | -0.504997776 | 2.79E-05 | 0.000778691 |
| GABRA4 | -0.702848859 | 2.81E-05 | 0.000780273 |
| CLDN11 | 0.623797481 | 2.81E-05 | 0.000780273 |
| FAM189A2 | 0.510492927 | 2.82E-05 | 0.00078149 |
| MT1F | 0.606965761 | 2.84E-05 | 0.000785939 |
| CPLX1 | -0.641680689 | 2.88E-05 | 0.000794698 |
| EGR1 | -0.634414315 | 2.92E-05 | 0.000801144 |
| SYT5 | -0.725422156 | 2.99E-05 | 0.000817889 |
| SCG3 | -0.563547137 | 3.02E-05 | 0.00082276 |
| VGF | -0.881967728 | 3.07E-05 | 0.0008261 |
| HSPB1 | 0.578448983 | 3.06E-05 | 0.0008261 |
| BEX5 | -0.843048749 | 3.11E-05 | 0.000833194 |
| PNMAL1 | -0.66983127 | 3.16E-05 | 0.000842869 |
| AASDHPPT | -0.570765103 | 3.17E-05 | 0.000844032 |
| RRAGA | -0.519501954 | 3.19E-05 | 0.00084781 |
| OCA2 | -0.773386334 | 3.29E-05 | 0.0008657 |
| ICAM5 | -0.52609669 | 3.31E-05 | 0.000868835 |
| EVI2A | 0.636100811 | 3.31E-05 | 0.000868835 |
| TNS1 | 0.522423728 | 3.34E-05 | 0.000872907 |
| MAP7D2 | -0.788875491 | 3.35E-05 | 0.000874455 |
| SLC27A4 | -0.575322507 | 3.35E-05 | 0.000874455 |
| SCN8A | -0.504198609 | 3.42E-05 | 0.000884375 |
| APOC1 | 0.583698855 | 3.46E-05 | 0.000891853 |
| NPY2R | -0.614892047 | 3.55E-05 | 0.000905888 |
| GUCY1B3 | -0.562693636 | 3.54E-05 | 0.000905888 |
| PCDH19 | -0.52785683 | 3.60E-05 | 0.000914605 |
| LOC81691 | -0.664859491 | 3.63E-05 | 0.00091939 |
| RTN4IP1 | -0.734984336 | 3.64E-05 | 0.000919891 |
| TXNIP | 0.874769849 | 3.66E-05 | 0.000921369 |
| PLP1 | 0.639140708 | 3.71E-05 | 0.000932764 |
| DOC2A | -0.734216188 | 3.75E-05 | 0.0009399 |
| IL10RA | 0.611207251 | 3.79E-05 | 0.00094872 |
| SNX10 | -0.66418607 | 3.79E-05 | 0.000948757 |
| MYOT | 0.668486815 | 3.82E-05 | 0.000951558 |
| SCG2 | -1.010968866 | 3.84E-05 | 0.000956395 |
| NETO2 | -0.619896642 | 3.87E-05 | 0.000961518 |
| REEP3 | 0.532492516 | 3.91E-05 | 0.000968818 |
| TRO | -0.543645233 | 3.92E-05 | 0.000969512 |
| NFKBIA | 0.533615746 | 4.03E-05 | 0.000989567 |
| LAIR1 | 0.560112236 | 4.03E-05 | 0.000989567 |
| EGR4 | -0.588278088 | 4.06E-05 | 0.000990028 |
| IFITM1 | 0.613324111 | 4.05E-05 | 0.000990028 |
| SH2D5 | -0.654154984 | 4.26E-05 | 0.001027071 |
| MAST3 | -0.526061395 | 4.34E-05 | 0.001038415 |
| PCCB | -0.518452636 | 4.37E-05 | 0.001040579 |
| CAPRIN2 | -0.503007858 | 4.36E-05 | 0.001040579 |
| TRIM22 | 0.687792414 | 4.38E-05 | 0.001040579 |
| AMN1 | -0.508973728 | 4.47E-05 | 0.001057477 |
| CIRBP | -0.522777986 | 4.48E-05 | 0.001058941 |
| SNX31 | 0.855588785 | 4.51E-05 | 0.001063399 |
| SYNGR3 | -0.718824254 | 4.58E-05 | 0.001071437 |
| SLC30A3 | -0.883179401 | 4.61E-05 | 0.001071685 |
| CCDC24 | -0.698917346 | 4.62E-05 | 0.001071685 |
| VRK1 | -0.592477131 | 4.60E-05 | 0.001071685 |
| ASB2 | -0.591096352 | 4.63E-05 | 0.001071685 |
| SCG5 | -0.860822676 | 4.75E-05 | 0.001088272 |
| ENO2 | -0.710074613 | 4.77E-05 | 0.001090518 |
| MB21D2 | -0.609274016 | 4.89E-05 | 0.001115359 |
| MEF2C | -0.64183317 | 4.91E-05 | 0.001117984 |
| DOK6 | -0.569109685 | 4.95E-05 | 0.001125031 |
| SNAP25 | -0.887713448 | 4.97E-05 | 0.001125983 |
| NECAB1 | -1.061718443 | 5.00E-05 | 0.001132023 |
| ATP6AP1 | -0.513871445 | 5.01E-05 | 0.001132101 |
| FUCA1 | -0.506860038 | 5.23E-05 | 0.001164244 |
| AP2M1 | -0.501095457 | 5.23E-05 | 0.001164244 |
| IFITM2 | 0.67050311 | 5.22E-05 | 0.001164244 |
| RFTN2 | 0.610209689 | 5.24E-05 | 0.001164847 |
| LY86-AS1 | -0.748701051 | 5.33E-05 | 0.001181434 |
| DDX1 | -0.510132719 | 5.35E-05 | 0.001185904 |
| KCNE4 | 0.827377124 | 5.37E-05 | 0.001186987 |
| SYBU | -0.624056639 | 5.39E-05 | 0.001188192 |
| YAP1 | 0.639119153 | 5.38E-05 | 0.001188192 |
| MDH1 | -0.895282539 | 5.52E-05 | 0.001212208 |
| DYNC1I1 | -0.831284857 | 5.52E-05 | 0.001212208 |
| ZBTB20 | 0.556267396 | 5.64E-05 | 0.001229479 |
| PRMT8 | -0.838298557 | 5.79E-05 | 0.001256764 |
| CADPS | -0.669438633 | 5.87E-05 | 0.001269578 |
| SEMA3A | -0.73089452 | 5.93E-05 | 0.001281445 |
| SYT13 | -0.732911566 | 5.97E-05 | 0.001287417 |
| FHOD3 | -0.565639527 | 6.10E-05 | 0.001308392 |
| TMEM158 | -0.629214333 | 6.15E-05 | 0.001315854 |
| SPHKAP | -0.895518036 | 6.17E-05 | 0.001317918 |
| FGF14 | -0.538371991 | 6.19E-05 | 0.001318668 |
| AP3B2 | -0.577397238 | 6.22E-05 | 0.001323631 |
| BAG3 | 0.657759366 | 6.27E-05 | 0.001332596 |
| PCP4L1 | -0.748738659 | 6.52E-05 | 0.001375785 |
| TEKT3 | -0.773149438 | 6.54E-05 | 0.001379195 |
| MSH2 | -0.666492053 | 6.59E-05 | 0.001384947 |
| CARD6 | 0.736409609 | 6.65E-05 | 0.001396439 |
| CHRNB2 | -0.503298817 | 6.68E-05 | 0.001397555 |
| CACNG1 | -0.701651383 | 6.78E-05 | 0.001416071 |
| VWDE | -0.636921629 | 6.81E-05 | 0.001419538 |
| PHYHIP | -0.613158018 | 6.90E-05 | 0.001434802 |
| COCH | -0.546866747 | 6.99E-05 | 0.001449226 |
| HLA-DPA1 | 0.618292116 | 7.04E-05 | 0.001457319 |
| HLF | -0.552202179 | 7.14E-05 | 0.001471736 |
| GSTO2 | -0.578748944 | 7.17E-05 | 0.0014747 |
| ACTN2 | -0.680075377 | 7.36E-05 | 0.001506608 |
| SERTAD1 | 0.523273075 | 7.36E-05 | 0.001506608 |
| GAD2 | -0.695816171 | 7.51E-05 | 0.001526989 |
| NLRP3 | -0.54675032 | 7.50E-05 | 0.001526989 |
| FGF1 | 0.559512857 | 7.57E-05 | 0.001535858 |
| CABP1 | -0.628779221 | 7.72E-05 | 0.001556693 |
| SSR4P1 | -0.67916837 | 7.82E-05 | 0.001570364 |
| PIM1 | 0.575984054 | 7.82E-05 | 0.001570364 |
| LRTM2 | -0.889701048 | 7.90E-05 | 0.001584626 |
| GLP2R | -0.704355565 | 7.93E-05 | 0.001588238 |
| PRKCB | -0.529523933 | 8.15E-05 | 0.001621277 |
| ZMAT4 | -0.751583225 | 8.34E-05 | 0.00164717 |
| CPNE9 | -0.649035787 | 8.35E-05 | 0.00164717 |
| HIGD1B | 0.8846757 | 8.34E-05 | 0.00164717 |
| GAS7 | -0.639660767 | 8.38E-05 | 0.001651924 |
| ACSS3 | 0.65332966 | 8.59E-05 | 0.001683515 |
| CD83 | -0.50879469 | 8.63E-05 | 0.00169043 |
| NT5DC3 | -0.582989315 | 8.74E-05 | 0.001707664 |
| SCN2B | -0.51292922 | 8.88E-05 | 0.001726323 |
| NDST3 | -0.630989266 | 8.95E-05 | 0.001734298 |
| LOC375196 | -0.676811298 | 9.02E-05 | 0.001746445 |
| EMP3 | 0.624308581 | 9.20E-05 | 0.001775427 |
| PARM1 | -0.737589133 | 9.22E-05 | 0.001775722 |
| TMEM67 | -0.501566996 | 9.25E-05 | 0.001779063 |
| ASPHD2 | -0.587896397 | 9.28E-05 | 0.001781528 |
| DOCK3 | -0.777459026 | 9.43E-05 | 0.001807025 |
| NRIP3 | -0.703688122 | 9.80E-05 | 0.001865255 |
| HTR7 | -0.509222397 | 9.84E-05 | 0.001867756 |
| LRFN2 | -0.623490863 | 9.93E-05 | 0.001882347 |
| EEF1A2 | -0.566601887 | 0.000102643 | 0.001920974 |
| NPTXR | -0.611576455 | 0.00010542 | 0.001965535 |
| MS4A6A | 0.680566978 | 0.000105524 | 0.001965535 |
| NMNAT2 | -0.828701918 | 0.000106332 | 0.00197122 |
| GABRG2 | -0.851471991 | 0.00010831 | 0.002003152 |
| PITHD1 | -0.595185478 | 0.000108832 | 0.002008678 |
| DUSP4 | -0.602940087 | 0.00011041 | 0.002032427 |
| FGF7 | -0.592630291 | 0.000115067 | 0.002103354 |
| RNF122 | 0.570072739 | 0.00011538 | 0.00210662 |
| TRIM47 | 0.506119162 | 0.000116029 | 0.002109491 |
| SENP8 | -0.549169163 | 0.00011684 | 0.002120948 |
| CDH18 | -0.56859684 | 0.000121554 | 0.002183795 |
| HS6ST2 | -0.58978445 | 0.000126284 | 0.002253316 |
| MLKL | 0.580971257 | 0.000126897 | 0.002261675 |
| EPDR1 | -0.709427688 | 0.000127427 | 0.002265977 |
| COPS4 | -0.509386883 | 0.000127416 | 0.002265977 |
| FBXO16 | -0.748369347 | 0.000128366 | 0.002280087 |
| NXF5 | -0.5604874 | 0.000131362 | 0.002317584 |
| FLT1 | 0.601212465 | 0.000132817 | 0.002335376 |
| MAPK6 | -0.504113511 | 0.000135098 | 0.002357005 |
| GRAMD3 | 0.628054996 | 0.000134994 | 0.002357005 |
| C1QB | 0.656754934 | 0.00013726 | 0.002386782 |
| FOSB | -0.873698111 | 0.000138587 | 0.002401773 |
| GRIN2A | -0.52224566 | 0.000138734 | 0.002401773 |
| NEK7 | 0.516675842 | 0.000139623 | 0.002406548 |
| STAB1 | 0.557455743 | 0.000141188 | 0.00242553 |
| TMOD1 | -0.504525783 | 0.000142567 | 0.002446537 |
| ACOT4 | -0.737311686 | 0.000145846 | 0.002489216 |
| GRM1 | -0.618652325 | 0.000148779 | 0.002529184 |
| SLITRK1 | -0.518990038 | 0.000150052 | 0.002543905 |
| PPM1E | -0.791125843 | 0.00015177 | 0.002560519 |
| PLK2 | -0.759692735 | 0.00015199 | 0.002560519 |
| RRAGB | -0.720159251 | 0.000155234 | 0.002595823 |
| RARRES1 | -0.614748884 | 0.000155214 | 0.002595823 |
| TM7SF2 | -0.576577735 | 0.000155741 | 0.002597575 |
| TOB1 | 0.514452575 | 0.000157657 | 0.002620743 |
| APOLD1 | 0.752160083 | 0.000159771 | 0.002649125 |
| SLC39A10 | -0.541100767 | 0.00016065 | 0.00265809 |
| PLSCR4 | 0.633408981 | 0.000160489 | 0.00265809 |
| TAS2R10 | -0.641814915 | 0.000163978 | 0.002698934 |
| ZNF214 | -0.60699855 | 0.000164801 | 0.002709631 |
| STXBP1 | -0.632358172 | 0.000165793 | 0.002723095 |
| ATP6V1B2 | -0.683529123 | 0.000168664 | 0.002755845 |
| DHRS11 | -0.512630454 | 0.00016886 | 0.002756167 |
| DCN | 0.509560584 | 0.000169615 | 0.002765624 |
| TRIP10 | 0.559230943 | 0.000171139 | 0.0027818 |
| KCNIP4 | -0.612084783 | 0.000174385 | 0.002831339 |
| TNFRSF10B | 0.515979826 | 0.00017524 | 0.002839653 |
| CSF1R | 0.582262687 | 0.000175669 | 0.002843657 |
| GJA4 | 0.670491429 | 0.000176105 | 0.002847785 |
| WBSCR17 | -0.651428419 | 0.000177855 | 0.002870175 |
| PARP14 | 0.549779791 | 0.000181582 | 0.002918317 |
| UBE2T | -0.732159904 | 0.000182239 | 0.002925887 |
| GRIA4 | -0.603742094 | 0.00018341 | 0.002941285 |
| EMP2 | 0.516904228 | 0.000185157 | 0.002960623 |
| ZDHHC23 | -0.514425323 | 0.000185687 | 0.002966081 |
| SLC5A3 | 0.512998038 | 0.00018808 | 0.002990401 |
| C1QTNF4 | -0.725922372 | 0.00019338 | 0.003054789 |
| ENTPD3 | -0.888958784 | 0.00019462 | 0.003068193 |
| CTSS | 0.504676508 | 0.000196228 | 0.003087342 |
| NIF3L1 | -0.573682785 | 0.000196963 | 0.003095814 |
| ERC2 | -0.594184652 | 0.000198522 | 0.00310787 |
| CYP26B1 | -0.594857457 | 0.000199118 | 0.0031141 |
| SYT4 | -0.878511778 | 0.000199914 | 0.00312034 |
| ELOVL4 | -0.63957137 | 0.000201161 | 0.003133558 |
| SYNPR | -0.866816239 | 0.000202258 | 0.003144413 |
| CNR1 | -0.740537683 | 0.00020668 | 0.003195686 |
| CBLN2 | -0.61549248 | 0.000206777 | 0.003195686 |
| GABRD | -0.740312692 | 0.000210229 | 0.003235295 |
| VIPR1 | -0.615916734 | 0.000215292 | 0.003304504 |
| CHAF1B | -0.689546813 | 0.000217561 | 0.003326329 |
| PPM1J | -0.689556364 | 0.000220181 | 0.003359841 |
| CDKN3 | -0.575470919 | 0.000221118 | 0.003367601 |
| DGKB | -0.521542359 | 0.000224461 | 0.003408738 |
| FGF13 | -0.557363427 | 0.000227642 | 0.003446901 |
| ATP6V1A | -0.516513238 | 0.00022961 | 0.003463541 |
| KIFAP3 | -0.6746813 | 0.000231474 | 0.003488103 |
| SLC2A13 | -0.609134666 | 0.000233406 | 0.003510142 |
| RAB6B | -0.542467092 | 0.000248825 | 0.003699865 |
| AMPH | -0.947861717 | 0.000251374 | 0.003727177 |
| OPCML | -0.518586736 | 0.000252191 | 0.003728727 |
| S100A10 | 0.534808223 | 0.00025198 | 0.003728727 |
| FGR | 0.682120938 | 0.000260904 | 0.003825125 |
| MOAP1 | -0.541861394 | 0.000262759 | 0.003848738 |
| NUDT18 | -0.541541098 | 0.000266608 | 0.003886979 |
| CPXM1 | -0.650079426 | 0.000270022 | 0.003929463 |
| LANCL1 | -0.50118777 | 0.00027467 | 0.003986791 |
| ITGA6 | 0.531296087 | 0.00028085 | 0.004054618 |
| ZNF540 | -0.514069999 | 0.000282064 | 0.004062403 |
| LEF1 | 0.505159431 | 0.000282543 | 0.004062715 |
| DAAM2 | 0.559760656 | 0.000284103 | 0.004081415 |
| LOC100131541 | 0.818620561 | 0.000286154 | 0.00410337 |
| CFI | 0.526403198 | 0.000291597 | 0.004173792 |
| SDR16C5 | -0.732371154 | 0.000305007 | 0.004293362 |
| FAM150B | -0.677458514 | 0.000304311 | 0.004293362 |
| SLIT2 | -0.5385788 | 0.000304679 | 0.004293362 |
| NPNT | 0.600641302 | 0.000303916 | 0.004293362 |
| MYO1F | 0.629406137 | 0.000303869 | 0.004293362 |
| CHI3L1 | 0.708882732 | 0.000303002 | 0.004293362 |
| ANGPT2 | 0.861772815 | 0.000302824 | 0.004293362 |
| C1orf145 | -0.583768195 | 0.000311028 | 0.004348849 |
| GRB14 | -0.589561165 | 0.000319194 | 0.004441407 |
| CHIC1 | -0.505982058 | 0.000319175 | 0.004441407 |
| HLA-DMB | 0.545258082 | 0.000319652 | 0.004441743 |
| UCHL1 | -0.682483193 | 0.000320647 | 0.004451639 |
| CP | 0.904579117 | 0.000323287 | 0.004476415 |
| CAP2 | -0.730478542 | 0.000329099 | 0.004532102 |
| IL1RL1 | 0.951216413 | 0.000336316 | 0.004612087 |
| NELL1 | -0.54188905 | 0.000338506 | 0.004638066 |
| LY86 | 0.522008305 | 0.000341029 | 0.004660439 |
| SPR | 0.514852344 | 0.000341995 | 0.004666229 |
| SLITRK4 | -0.647437039 | 0.000344307 | 0.00468891 |
| CDH19 | 0.531304475 | 0.00034643 | 0.004701502 |
| C3orf80 | -0.798645123 | 0.000349632 | 0.004736769 |
| KRT222 | -0.666356485 | 0.000351911 | 0.004763537 |
| BTN3A1 | 0.604184329 | 0.000352249 | 0.004764015 |
| BAZ1A | 0.504841295 | 0.000354239 | 0.004782682 |
| CASP7 | 0.550977608 | 0.000355175 | 0.004790647 |
| RASGRF1 | -0.502422944 | 0.000359689 | 0.004827207 |
| TMEM130 | -0.59300738 | 0.000363482 | 0.004859616 |
| C3 | 0.553462912 | 0.000364971 | 0.004864837 |
| ARNTL2 | -0.500189616 | 0.000377583 | 0.004986388 |
| MYT1L | -0.709421547 | 0.000385649 | 0.005071576 |
| CREG2 | -0.676379557 | 0.000386308 | 0.005075984 |
| ELAVL4 | -0.681307027 | 0.000387698 | 0.005089997 |
| CD99 | 0.520168203 | 0.000392744 | 0.005116782 |
| HTR7P1 | -0.571845091 | 0.000397929 | 0.005172417 |
| HMOX1 | 0.617372883 | 0.000397721 | 0.005172417 |
| OLFM3 | -0.830661851 | 0.000401446 | 0.005209505 |
| GAL3ST4 | 0.517855503 | 0.000407154 | 0.005253192 |
| NACC2 | 0.519155291 | 0.000411722 | 0.00529944 |
| TNFRSF10D | 0.680404415 | 0.000411751 | 0.00529944 |
| SLC14A1 | 0.928144939 | 0.000420293 | 0.005382901 |
| RBM3 | -0.698084055 | 0.000423214 | 0.005402694 |
| IFITM3 | 0.5544281 | 0.000422917 | 0.005402694 |
| CCKBR | -0.606652178 | 0.00042609 | 0.005421776 |
| GFRA2 | -0.575231111 | 0.000443927 | 0.005616873 |
| TLR4 | 0.545719899 | 0.000444926 | 0.005624987 |
| BEX1 | -0.672475897 | 0.000450473 | 0.005689003 |
| PDP1 | -0.533928465 | 0.000451077 | 0.005689003 |
| GRIN3A | -0.655192216 | 0.000469512 | 0.005879005 |
| KCNC2 | -0.728772432 | 0.00047697 | 0.00594396 |
| ID3 | 0.629712004 | 0.000485396 | 0.006035371 |
| LAMB2 | 0.50197088 | 0.000493421 | 0.006100545 |
| MBLAC2 | -0.516283735 | 0.000494877 | 0.006113734 |
| KCNS1 | -0.642421372 | 0.000502117 | 0.00617403 |
| C5orf22 | -0.558561422 | 0.000502799 | 0.006177586 |
| SLC7A2 | 0.523404318 | 0.000507371 | 0.006204625 |
| LY6D | -0.698113127 | 0.000511758 | 0.00623884 |
| ELOVL7 | 0.539840873 | 0.000511605 | 0.00623884 |
| SLC16A1 | 0.541577954 | 0.000520027 | 0.006324914 |
| SLC17A6 | -0.738610057 | 0.000533008 | 0.006447838 |
| PCDH8 | -0.860218171 | 0.000549678 | 0.006623983 |
| RRAS | 0.507200096 | 0.000559191 | 0.00670773 |
| SIDT1 | -0.631461733 | 0.00056438 | 0.00675964 |
| PKIB | -0.529279177 | 0.000567084 | 0.006786855 |
| KCNG3 | -0.559136534 | 0.000576228 | 0.006880557 |
| ZNF391 | -0.545746368 | 0.000576172 | 0.006880557 |
| NECAP1 | -0.525793495 | 0.000579627 | 0.006915887 |
| TAGLN2 | 0.50709603 | 0.000592039 | 0.007042571 |
| ARPP21 | -0.532547559 | 0.000600506 | 0.007132479 |
| RBM11 | -0.658886978 | 0.000611146 | 0.007231076 |
| CALM1 | -0.678549455 | 0.000613514 | 0.007237719 |
| STON2 | 0.583213176 | 0.000615821 | 0.007259478 |
| HCLS1 | 0.643239222 | 0.000617222 | 0.00726671 |
| NME5 | -0.553476167 | 0.00062159 | 0.007305538 |
| ZNF57 | -0.503478452 | 0.000626864 | 0.007351024 |
| C10orf10 | 0.650852179 | 0.000638044 | 0.007459841 |
| HVCN1 | 0.562666621 | 0.000644993 | 0.007513027 |
| ADRA1B | -0.548145883 | 0.000664625 | 0.007679129 |
| VSTM2A | -0.598614902 | 0.000665863 | 0.00768756 |
| FAT1 | 0.500676335 | 0.000666334 | 0.00768756 |
| PAX7 | -0.508266063 | 0.000670179 | 0.00772625 |
| ACSL5 | 0.557472066 | 0.000675684 | 0.007783997 |
| ID4 | 0.52384023 | 0.000677582 | 0.007799289 |
| ACOT7 | -0.675472672 | 0.000685779 | 0.007827442 |
| C21orf62 | 0.594372317 | 0.000684594 | 0.007827442 |
| CNTNAP2 | -0.554098073 | 0.000706573 | 0.008051236 |
| SLC16A14 | -0.559074013 | 0.000711887 | 0.008088311 |
| BEX2 | -0.540743384 | 0.000713655 | 0.008095171 |
| CALY | -0.586202661 | 0.000718572 | 0.008113527 |
| TMEM151A | -0.596922963 | 0.000742672 | 0.008323656 |
| CPNE4 | -0.806571474 | 0.000746948 | 0.008357264 |
| MS4A4A | 0.636808563 | 0.000756474 | 0.008436189 |
| COLEC12 | 0.620858427 | 0.000758793 | 0.008450045 |
| PP12719 | 0.535619508 | 0.000767156 | 0.008512988 |
| HTR2C | -0.644810931 | 0.000797359 | 0.008787632 |
| SRSF12 | -0.517013082 | 0.000821269 | 0.008965452 |
| PNMA6A | -0.567605308 | 0.000837688 | 0.009108988 |
| MUM1L1 | -0.632812914 | 0.000841917 | 0.009123385 |
| MOXD1 | -0.507677352 | 0.00084674 | 0.009156687 |
| PIP5K1B | -0.502849749 | 0.000860667 | 0.009268996 |
| ALB | -0.511247773 | 0.000862912 | 0.009286798 |
| SUSD1 | -0.528801046 | 0.000865453 | 0.009307772 |
| PGF | 0.504567675 | 0.000880696 | 0.00942894 |
| FNDC9 | -0.60008605 | 0.000889864 | 0.009492283 |
| DNAH2 | -0.566107 | 0.000917474 | 0.009720767 |
| IL1RL2 | -0.625193414 | 0.000931461 | 0.009852421 |
| GSTZ1 | -0.574881208 | 0.000931782 | 0.009852421 |
| SERPINI1 | -0.561961982 | 0.000932978 | 0.009858422 |
| TLR5 | 0.556167138 | 0.000947923 | 0.009989457 |
| LRRC29 | -0.646047443 | 0.000953873 | 0.010045413 |
| STAR | -0.665512461 | 0.000957272 | 0.01006771 |
| PLEKHH1 | 0.58538104 | 0.000982451 | 0.010275723 |
| SLC32A1 | -0.657831034 | 0.000998349 | 0.010402178 |
| GABRA1 | -0.840243776 | 0.001020255 | 0.010594723 |
| CD24 | -0.530293929 | 0.001034812 | 0.010725164 |
| RTN1 | -0.710436682 | 0.001036254 | 0.010733025 |
| MOBP | 0.597452823 | 0.001052317 | 0.010877867 |
| TMEM200A | -0.651892191 | 0.001056213 | 0.01091096 |
| KCNJ6 | -0.698361039 | 0.001067592 | 0.010985365 |
| GLIPR1 | -0.595781307 | 0.001071714 | 0.011005917 |
| KLK7 | -0.667222101 | 0.001072977 | 0.011011685 |
| TSPO | 0.524164711 | 0.001076612 | 0.011040171 |
| C1orf87 | 0.703987593 | 0.001077862 | 0.011040171 |
| KCNB2 | -0.571225258 | 0.001098745 | 0.011224787 |
| F13A1 | 0.697021533 | 0.001110674 | 0.011302527 |
| SCN9A | -0.519201711 | 0.001153834 | 0.011636032 |
| LIPC | -0.507149975 | 0.001153855 | 0.011636032 |
| MCF2 | -0.615395081 | 0.001158547 | 0.011658232 |
| IL13RA2 | -0.734653082 | 0.001172663 | 0.011750576 |
| UNC13A | -0.564250385 | 0.001173602 | 0.011752485 |
| PIM2 | -0.539262219 | 0.001181079 | 0.01180478 |
| EFNB3 | -0.511883043 | 0.001193651 | 0.011892583 |
| TGFBI | 0.694572539 | 0.001213303 | 0.012057777 |
| GPR61 | -0.616659386 | 0.001225476 | 0.012155667 |
| FCGR2A | 0.537012408 | 0.001261998 | 0.012470673 |
| NXPH1 | -0.504836503 | 0.001273412 | 0.012543989 |
| ITGA10 | 0.516823472 | 0.001283074 | 0.012599644 |
| PLP2 | 0.524916817 | 0.001282402 | 0.012599644 |
| BTN3A2 | 0.50911423 | 0.00131591 | 0.01280993 |
| TM4SF1 | 0.500590037 | 0.001336863 | 0.012965676 |
| TCL1B | 0.578215215 | 0.001369031 | 0.013228631 |
| DLX6 | -0.596857826 | 0.001373503 | 0.01325553 |
| C11orf96 | 0.631351521 | 0.001391645 | 0.013356734 |
| RNF165 | -0.500394323 | 0.001418448 | 0.013547729 |
| CCK | -0.77678537 | 0.001433643 | 0.013659628 |
| MAEL | -0.647162758 | 0.001433449 | 0.013659628 |
| SMPX | -0.753799296 | 0.001458095 | 0.013840062 |
| MPZL2 | 0.543005145 | 0.001458751 | 0.013840062 |
| CHGA | -0.652488024 | 0.001464945 | 0.013882058 |
| XK | -0.609016608 | 0.001482492 | 0.014006067 |
| TIMP1 | 0.593297075 | 0.00155204 | 0.014445841 |
| BTBD11 | -0.634020494 | 0.001555952 | 0.01445486 |
| LCP1 | 0.578928965 | 0.001730663 | 0.015717103 |
| CDKN1A | 0.51301226 | 0.001743792 | 0.015799778 |
| CHST6 | 0.601602246 | 0.001742999 | 0.015799778 |
| RNASE2 | 0.7636829 | 0.001748937 | 0.015828129 |
| ADAMTS3 | -0.58489068 | 0.001763656 | 0.015897203 |
| GLS2 | -0.601517011 | 0.001789745 | 0.016049445 |
| MAP2K1 | -0.544109012 | 0.001804474 | 0.016153847 |
| PRMT6 | -0.537337047 | 0.001809027 | 0.01618538 |
| KIAA1107 | -0.553534691 | 0.001813323 | 0.016205349 |
| HS3ST2 | -0.742729952 | 0.001834967 | 0.016370824 |
| ITGB4 | 0.504028511 | 0.001866789 | 0.016601329 |
| SYCE1 | -0.540442126 | 0.001907144 | 0.016796706 |
| FHL2 | -0.528219492 | 0.001905649 | 0.016796706 |
| KDM4D | -0.573651074 | 0.001955706 | 0.017088752 |
| RASL12 | 0.59513238 | 0.002020255 | 0.017525992 |
| GPR22 | -0.677373561 | 0.002024324 | 0.017532496 |
| ROBO2 | -0.524893542 | 0.002116306 | 0.018107088 |
| C3AR1 | 0.559433283 | 0.002130569 | 0.018142186 |
| CCNA1 | -0.572016014 | 0.002174171 | 0.018413576 |
| PHYHD1 | 0.61740507 | 0.00217534 | 0.018413576 |
| ARMC3 | 0.512962962 | 0.002190727 | 0.018523017 |
| PCSK2 | -0.542674802 | 0.0022313 | 0.018805376 |
| CHRDL2 | -0.54063441 | 0.002245406 | 0.018884506 |
| NUDT11 | -0.65632695 | 0.002268917 | 0.019041438 |
| SH3GL2 | -0.727480893 | 0.002298329 | 0.019226616 |
| RGS1 | 0.843301684 | 0.0023005 | 0.019234536 |
| WIF1 | -0.740775831 | 0.002303958 | 0.01925319 |
| EGR2 | -0.622185539 | 0.002367072 | 0.019613596 |
| PPP1R32 | -0.515089716 | 0.002377159 | 0.019666041 |
| PDLIM1 | 0.632714299 | 0.002421942 | 0.019931518 |
| CTXN3 | -0.848858862 | 0.002457313 | 0.020169746 |
| FMO2 | 0.551467046 | 0.002514197 | 0.020541684 |
| SERPINH1 | 0.642813641 | 0.002507231 | 0.020541684 |
| DACH2 | -0.654269929 | 0.002574932 | 0.020905679 |
| SLC1A6 | -0.542958764 | 0.002590227 | 0.020975626 |
| SLC7A4 | -0.516006428 | 0.002668439 | 0.021465077 |
| IL4R | 0.636170823 | 0.002742993 | 0.021941149 |
| PNCK | -0.576703371 | 0.002918923 | 0.022939404 |
| LINC00326 | -0.608567506 | 0.002930869 | 0.022975788 |
| CDH7 | -0.517228925 | 0.002965907 | 0.023181481 |
| GIMAP7 | 0.544153273 | 0.002992311 | 0.023294628 |
| PAK3 | -0.548442258 | 0.003020395 | 0.023478335 |
| DDIT4 | 0.535523798 | 0.003159121 | 0.024256487 |
| TAAR5 | -0.594093491 | 0.003177036 | 0.024334544 |
| DGAT2 | -0.556337018 | 0.003184703 | 0.024357627 |
| SOCS3 | 0.704874974 | 0.00331595 | 0.025177492 |
| GPR4 | 0.58483914 | 0.003360456 | 0.025417091 |
| CXCL1 | 0.573087582 | 0.003391291 | 0.025600989 |
| S100A11 | 0.555791196 | 0.003419408 | 0.025726665 |
| HLA-DMA | 0.535701325 | 0.003448169 | 0.025881045 |
| TC2N | -0.530338419 | 0.003477785 | 0.026053521 |
| TRIM54 | -0.543302066 | 0.003481165 | 0.026066403 |
| CFH | 0.663019266 | 0.003580188 | 0.026542076 |
| SLC6A12 | 0.546408252 | 0.003724809 | 0.027338639 |
| TMEM17 | -0.547464962 | 0.00375373 | 0.027516836 |
| LDHC | -0.571133308 | 0.003784356 | 0.027676741 |
| ZNF385B | -0.582375298 | 0.003848141 | 0.028023438 |
| SLCO4A1 | 0.587516314 | 0.003861955 | 0.028087272 |
| PTPN5 | -0.556107956 | 0.003975016 | 0.028723286 |
| PKP3 | -0.527374119 | 0.004061645 | 0.029181988 |
| ZFP36 | 0.60642549 | 0.004214031 | 0.029967712 |
| PNMA2 | -0.546892633 | 0.004265367 | 0.030168822 |
| SERTM1 | -0.580660455 | 0.004477023 | 0.031215906 |
| C5AR1 | 0.662470713 | 0.004676142 | 0.032238436 |
| NEFM | -0.592338796 | 0.004699278 | 0.032348995 |
| TAC1 | -0.911861653 | 0.004707973 | 0.032380458 |
| NAP1L2 | -0.586532983 | 0.004715473 | 0.032394443 |
| SLC22A9 | -0.527899529 | 0.005022464 | 0.033904872 |
| PLA1A | 0.703228391 | 0.005361432 | 0.035522621 |
| GALNTL5 | -0.590785004 | 0.005442277 | 0.035935504 |
| NMU | -0.746119269 | 0.005625738 | 0.036883228 |
| XAGE-4 | 0.632836595 | 0.005628713 | 0.036886331 |
| VSTM1 | -0.669135417 | 0.005695239 | 0.037245581 |
| NLGN4Y | -0.688297454 | 0.005769128 | 0.037587901 |
| PTPRT | -0.502874058 | 0.005958009 | 0.038483199 |
| GJA1 | 0.501157863 | 0.006079159 | 0.039059595 |
| CYP4X1 | -0.548326403 | 0.006168819 | 0.039407159 |
| FBXO40 | -0.587013256 | 0.006189202 | 0.039473133 |
| CDH8 | -0.590495659 | 0.006201653 | 0.039536482 |
| PRSS35 | -0.691039904 | 0.006294543 | 0.04003115 |
| PIRT | 0.509735039 | 0.00640788 | 0.040571182 |
| SYT16 | -0.520695094 | 0.006485922 | 0.040955844 |
| PRKX | 0.531052431 | 0.006530406 | 0.041147845 |
| SOSTDC1 | -0.817106372 | 0.006542562 | 0.041191385 |
| DYDC2 | -0.637227248 | 0.006620862 | 0.041551068 |
| VIP | -0.566655173 | 0.006719622 | 0.041994933 |
| GRP | -0.564397634 | 0.00687577 | 0.04279165 |
| HTR3B | -0.556656051 | 0.007846012 | 0.047438613 |
| PDYN | -0.639542926 | 0.007869054 | 0.04754132 |
| SUN3 | -0.548684414 | 0.008006603 | 0.048279447 |
| TMEM169 | -0.524733502 | 0.008147939 | 0.048940886 |
| FOS | 0.600730361 | 0.008215397 | 0.049217155 |
| HTR5A | -0.522190746 | 0.008254784 | 0.049377762 |
| CAPZA3 | -0.557233019 | 0.008271687 | 0.049422384 |
| MRAP2 | -0.680601303 | 0.008278801 | 0.049446076 |

Note: FDR, false discovery rate.

**Supplementary Table 3. Genes in each module.**

| **Module** | **Genes** |
| --- | --- |
| Black | BTN3A2 |
|  | C1QB |
|  | C1QC |
|  | C3 |
|  | C3AR1 |
|  | CCR1 |
|  | CD163 |
|  | CSF1R |
|  | CXCL16 |
|  | FCGBP |
|  | FCGR2A |
|  | GAL3ST4 |
|  | HCLS1 |
|  | HLA-DMA |
|  | HLA-DMB |
|  | HLA-DPA1 |
|  | IL10RA |
|  | LCP1 |
|  | LY6D |
|  | MS4A4A |
|  | MS4A6A |
|  | RGS1 |
|  | RHBDF2 |
|  | SCIN |
|  | TRIM22 |
|  | VSIG4 |
| Blue | ADAMTS1 |
|  | AEBP1 |
|  | APLNR |
|  | APOLD1 |
|  | B2M |
|  | BACE2 |
|  | BAG3 |
|  | C10orf10 |
|  | CD99 |
|  | CDKN1A |
|  | CFH |
|  | CHI3L1 |
|  | CHST6 |
|  | CLIC1 |
|  | COLEC12 |
|  | DCN |
|  | DTX3L |
|  | ELOVL7 |
|  | EMP1 |
|  | EMP3 |
|  | EPS8 |
|  | FOS |
|  | GBP2 |
|  | GEM |
|  | GFAP |
|  | GPR4 |
|  | HIGD1B |
|  | HSPB1 |
|  | IFITM1 |
|  | IFITM2 |
|  | IFITM3 |
|  | ITPKB |
|  | LAIR1 |
|  | LAMB2 |
|  | LEF1 |
|  | NACC2 |
|  | PDLIM1 |
|  | PECAM1 |
|  | PFKFB3 |
|  | RASL12 |
|  | RHOJ |
|  | S100A10 |
|  | SERPINA3 |
|  | SERTAD1 |
|  | SLC4A11 |
|  | SLC5A3 |
|  | SLC6A12 |
|  | SPR |
|  | SRGN |
|  | TAGLN2 |
|  | TIMP1 |
|  | TNFAIP3 |
|  | TNFRSF1A |
|  | TRIM47 |
|  | TRIP10 |
|  | ZFP36 |
| Brown | ACTR10 |
|  | ADAMTS3 |
|  | ARHGDIG |
|  | BDNF |
|  | BTBD11 |
|  | C1orf87 |
|  | C1R |
|  | CACNG1 |
|  | CCDC113 |
|  | CDK5 |
|  | CHRNB2 |
|  | CLMN |
|  | CMTM3 |
|  | COCH |
|  | CXCL1 |
|  | DGAT2 |
|  | EMP2 |
|  | GOLT1A |
|  | GPR61 |
|  | GSTZ1 |
|  | HTR2C |
|  | HTR3B |
|  | HTR7 |
|  | ICAM5 |
|  | IL1RL2 |
|  | INPP5D |
|  | LOC151760 |
|  | LOC81691 |
|  | NLRP3 |
|  | NMU |
|  | NPNT |
|  | NRN1 |
|  | OCA2 |
|  | PART1 |
|  | PHYHD1 |
|  | PI4KA |
|  | PIM2 |
|  | PKP3 |
|  | PLK2 |
|  | PPME1 |
|  | RAB3A |
|  | SCN9A |
|  | SDR16C5 |
|  | SLC1A6 |
|  | SLC27A4 |
|  | SLC7A7 |
|  | STMN1 |
|  | SYCE1 |
|  | SYT16 |
|  | TAAR5 |
|  | TAS2R10 |
|  | TMEM59L |
|  | TMEM67 |
|  | VASP |
|  | ZNF214 |
| Green | AP3B2 |
|  | ATP8A2 |
|  | CAMKK2 |
|  | CAPRIN2 |
|  | CDH18 |
|  | CHGB |
|  | CLSTN1 |
|  | CPLX1 |
|  | CRYM |
|  | DYDC2 |
|  | GABRA1 |
|  | GABRD |
|  | GAS7 |
|  | GPR158 |
|  | GRIA4 |
|  | KIAA0513 |
|  | MAL2 |
|  | NDRG4 |
|  | NPTX1 |
|  | NRIP3 |
|  | NRXN3 |
|  | PHYHIP |
|  | RASGRF1 |
|  | RPH3A |
|  | SCN2A |
|  | SCN2B |
|  | SEZ6L2 |
|  | SLC8A2 |
|  | STMN2 |
|  | SV2B |
|  | TERF2IP |
| Grey | ACOT4 |
|  | ALB |
|  | ANKRD16 |
|  | ARMC3 |
|  | ARNTL2 |
|  | ASB2 |
|  | B9D1 |
|  | BOK |
|  | BTN3A1 |
|  | C14orf79 |
|  | C21orf62 |
|  | C5AR1 |
|  | CALY |
|  | CAPZA3 |
|  | CARD6 |
|  | CDH8 |
|  | CHAF1B |
|  | CHRDL2 |
|  | COL24A1 |
|  | COX18 |
|  | CPNE9 |
|  | CRH |
|  | CRMP1 |
|  | CTSS |
|  | CXCR4 |
|  | DLX6 |
|  | DOC2A |
|  | DOCK3 |
|  | DUSP4 |
|  | EGR2 |
|  | EGR4 |
|  | F13A1 |
|  | FAM150B |
|  | FBXO16 |
|  | FBXO4 |
|  | FBXO40 |
|  | FGF14 |
|  | FUCA1 |
|  | FXYD7 |
|  | GALNTL5 |
|  | GJC1 |
|  | GRP |
|  | HMOX1 |
|  | IDH3G |
|  | IGF1 |
|  | IL13RA2 |
|  | IL1RL1 |
|  | KDM4D |
|  | KLHDC9 |
|  | LDHC |
|  | LIPC |
|  | LRTM2 |
|  | LY86 |
|  | MBOAT7 |
|  | MET |
|  | NLGN4Y |
|  | NPTXR |
|  | NPY2R |
|  | NXF5 |
|  | OLFM3 |
|  | OSCP1 |
|  | PAX7 |
|  | PCDH8 |
|  | PCP4L1 |
|  | PGF |
|  | PIM1 |
|  | PLP2 |
|  | PNOC |
|  | PPEF1 |
|  | PPM1J |
|  | PRKCG |
|  | PSMA1 |
|  | RAB3B |
|  | RARRES1 |
|  | REEP3 |
|  | RGS17 |
|  | RNF165 |
|  | RWDD2B |
|  | S100A11 |
|  | SLC9A7 |
|  | SMPX |
|  | SNX31 |
|  | STAB1 |
|  | STAR |
|  | SYT5 |
|  | TASP1 |
|  | TEKT3 |
|  | TGFBI |
|  | TLR5 |
|  | TM7SF2 |
|  | TMEM200A |
|  | TNFRSF10D |
|  | TRIM54 |
|  | TTLL1 |
|  | TXNDC9 |
|  | UNC13A |
|  | VWDE |
|  | WDR86 |
|  | XAGE-4 |
|  | ZBBX |
| Red | ANLN |
|  | ANP32B |
|  | APOC1 |
|  | AZGP1 |
|  | BAZ1A |
|  | BCAS1 |
|  | CDH19 |
|  | CDKN3 |
|  | CLDN11 |
|  | DAAM2 |
|  | ERMN |
|  | EVI2A |
|  | FAM107B |
|  | FGF1 |
|  | HSPA2 |
|  | KIF1C |
|  | LPAR1 |
|  | MOBP |
|  | NEK7 |
|  | NMNAT2 |
|  | PIP5K1B |
|  | PLEKHH1 |
|  | PLP1 |
|  | QKI |
|  | SEPP1 |
|  | SLA |
|  | SPP1 |
|  | TSPO |
|  | UGT8 |
|  | ZNF57 |
| Turquoise | AASDHPPT |
|  | ACOT7 |
|  | ACSL5 |
|  | ACTN2 |
|  | ADAM23 |
|  | ADCYAP1 |
|  | ADRA1B |
|  | AKAP5 |
|  | AMN1 |
|  | AMPH |
|  | ANGPT2 |
|  | ANO3 |
|  | AP2M1 |
|  | APOO |
|  | ARPC1B |
|  | ASPHD2 |
|  | ASTN1 |
|  | ATCAY |
|  | ATL1 |
|  | ATP5A1 |
|  | ATP5B |
|  | ATP6AP1 |
|  | ATP6V1A |
|  | ATP6V1B2 |
|  | ATP6V1C1 |
|  | ATP6V1E1 |
|  | ATRNL1 |
|  | BASP1 |
|  | BCAS2 |
|  | BCL6 |
|  | BEND5 |
|  | BEX1 |
|  | BEX2 |
|  | BEX5 |
|  | BFSP1 |
|  | BSN |
|  | BST2 |
|  | C1QTNF4 |
|  | C2orf80 |
|  | C3orf14 |
|  | C5orf22 |
|  | CABP1 |
|  | CACNB3 |
|  | CACNG3 |
|  | CADPS |
|  | CALM1 |
|  | CAMK1G |
|  | CAMK2D |
|  | CAMKV |
|  | CAP2 |
|  | CARTPT |
|  | CASP7 |
|  | CBLN2 |
|  | CBLN4 |
|  | CCDC24 |
|  | CCK |
|  | CCKBR |
|  | CCNA1 |
|  | CCNH |
|  | CCT6B |
|  | CD200 |
|  | CD24 |
|  | CD83 |
|  | CDH10 |
|  | CDH7 |
|  | CECR6 |
|  | CFI |
|  | CHCHD6 |
|  | CHGA |
|  | CHIC1 |
|  | CHMP1B |
|  | CHN1 |
|  | CHRM1 |
|  | CHRM3 |
|  | CIRBP |
|  | CISD1 |
|  | CITED1 |
|  | CLIP3 |
|  | CNR1 |
|  | CNTNAP2 |
|  | CNTNAP5 |
|  | COG1 |
|  | COPS4 |
|  | COPS7A |
|  | CP |
|  | CPNE4 |
|  | CPXM1 |
|  | CREG2 |
|  | CRYAB |
|  | CTXN3 |
|  | CYFIP2 |
|  | CYP26B1 |
|  | CYP4X1 |
|  | DACH2 |
|  | DCLK1 |
|  | DDAH1 |
|  | DDIT4 |
|  | DDN |
|  | DDX1 |
|  | DDX25 |
|  | DDX28 |
|  | DGKB |
|  | DGKI |
|  | DHRS11 |
|  | DIRAS1 |
|  | DLG3 |
|  | DLGAP2 |
|  | DNAH1 |
|  | DNAH2 |
|  | DNAJC19 |
|  | DNM1L |
|  | DOK6 |
|  | DYNC1I1 |
|  | EEF1A2 |
|  | EFNB3 |
|  | EGR1 |
|  | EHD3 |
|  | ELAVL4 |
|  | ELOVL4 |
|  | ENC1 |
|  | ENO2 |
|  | ENTPD3 |
|  | ENTPD6 |
|  | EPDR1 |
|  | EPHA5 |
|  | ERC2 |
|  | EVI2B |
|  | FABP3 |
|  | FAM102B |
|  | FAM19A1 |
|  | FAM49A |
|  | FAR2 |
|  | FBXL16 |
|  | FBXO34 |
|  | FBXW7 |
|  | FGF12 |
|  | FGF13 |
|  | FGF7 |
|  | FGR |
|  | FH |
|  | FHL2 |
|  | FHOD3 |
|  | FIBP |
|  | FIG4 |
|  | FKBP1B |
|  | FLT1 |
|  | FMO2 |
|  | FOSB |
|  | FRMPD4 |
|  | FSTL4 |
|  | GABBR2 |
|  | GABRA4 |
|  | GABRA5 |
|  | GABRB3 |
|  | GABRG2 |
|  | GAD1 |
|  | GAD2 |
|  | GAP43 |
|  | GARS |
|  | GDA |
|  | GFRA2 |
|  | GIMAP7 |
|  | GJA4 |
|  | GLIPR1 |
|  | GLMN |
|  | GLP2R |
|  | GLS2 |
|  | GMPR |
|  | GNG2 |
|  | GNG3 |
|  | GNPDA2 |
|  | GOT1 |
|  | GOT2 |
|  | GPATCH2 |
|  | GPHN |
|  | GPR22 |
|  | GPR88 |
|  | GPRASP1 |
|  | GRB14 |
|  | GRIN2A |
|  | GRIN2B |
|  | GRIN3A |
|  | GRM1 |
|  | GSS |
|  | GSTO2 |
|  | GUCY1B3 |
|  | GULP1 |
|  | HAPLN1 |
|  | HLF |
|  | HMGCS1 |
|  | HPCA |
|  | HPRT1 |
|  | HS3ST2 |
|  | HS6ST2 |
|  | HS6ST3 |
|  | HSPB3 |
|  | HTR2A |
|  | HTR5A |
|  | HVCN1 |
|  | ICA1 |
|  | ID3 |
|  | IFI16 |
|  | IL4R |
|  | IMPAD1 |
|  | INA |
|  | ITFG1 |
|  | ITGA10 |
|  | ITGB4 |
|  | JAKMIP1 |
|  | KCNB2 |
|  | KCNC2 |
|  | KCNE4 |
|  | KCNF1 |
|  | KCNG3 |
|  | KCNIP4 |
|  | KCNJ6 |
|  | KCNQ3 |
|  | KCNS1 |
|  | KCNS2 |
|  | KCNV1 |
|  | KCTD1 |
|  | KIAA1107 |
|  | KIAA1644 |
|  | KIFAP3 |
|  | KLK7 |
|  | KRT222 |
|  | LANCL1 |
|  | LETMD1 |
|  | LHFPL4 |
|  | LIN7B |
|  | LINGO1 |
|  | LINGO2 |
|  | LMBRD2 |
|  | LNX1 |
|  | LOC100131541 |
|  | LRFN2 |
|  | LRRC29 |
|  | LRRC7 |
|  | LRRTM1 |
|  | MAEL |
|  | MAFF |
|  | MAGED1 |
|  | MAP2 |
|  | MAP2K1 |
|  | MAP7D2 |
|  | MAPK6 |
|  | MAPK9 |
|  | MAST3 |
|  | MBLAC2 |
|  | MCF2 |
|  | MDH1 |
|  | MDH2 |
|  | MECR |
|  | MEF2C |
|  | MEST |
|  | MID1IP1 |
|  | MKKS |
|  | MLKL |
|  | MLLT11 |
|  | MOAP1 |
|  | MOXD1 |
|  | MPZL2 |
|  | MRAP2 |
|  | MRPL15 |
|  | MSH2 |
|  | MTCH2 |
|  | MTX2 |
|  | MUM1L1 |
|  | MYL5 |
|  | MYO1F |
|  | MYOT |
|  | MYT1L |
|  | NAP1L2 |
|  | NAP1L5 |
|  | NAPB |
|  | NCALD |
|  | NDST3 |
|  | NDUFA9 |
|  | NECAB1 |
|  | NECAB2 |
|  | NECAP1 |
|  | NEFM |
|  | NELL1 |
|  | NELL2 |
|  | NETO2 |
|  | NEUROD6 |
|  | NFKBIA |
|  | NGEF |
|  | NIF3L1 |
|  | NIPAL2 |
|  | NIT2 |
|  | NME5 |
|  | NNAT |
|  | NPTX2 |
|  | NRGN |
|  | NRSN1 |
|  | NUDT11 |
|  | NUDT18 |
|  | NUDT7 |
|  | NUPR1 |
|  | NXPH1 |
|  | OPCML |
|  | OPN3 |
|  | OXCT1 |
|  | P4HTM |
|  | PACSIN1 |
|  | PAK3 |
|  | PARM1 |
|  | PARP14 |
|  | PARP2 |
|  | PCCB |
|  | PCDH19 |
|  | PCSK1 |
|  | PCSK2 |
|  | PCYOX1L |
|  | PDE1A |
|  | PDHB |
|  | PDK3 |
|  | PDYN |
|  | PFN2 |
|  | PGM2L1 |
|  | PGRMC1 |
|  | PHYHIPL |
|  | PIAS2 |
|  | PIRT |
|  | PKIB |
|  | PKNOX2 |
|  | PLA1A |
|  | PLD3 |
|  | PLSCR4 |
|  | PNCK |
|  | PNMA2 |
|  | PNMA6A |
|  | PNMAL1 |
|  | PPIA |
|  | PPM1E |
|  | PPP1R14C |
|  | PPP4R4 |
|  | PRKCB |
|  | PRMT6 |
|  | PRMT8 |
|  | PRSS35 |
|  | PTDSS1 |
|  | PTK2B |
|  | PTPN3 |
|  | PTPN5 |
|  | PTPRT |
|  | RAB15 |
|  | RAB27B |
|  | RAB3C |
|  | RAB6B |
|  | RASAL1 |
|  | RASL10A |
|  | RBM11 |
|  | RBM3 |
|  | RBP4 |
|  | RCAN2 |
|  | REEP1 |
|  | RFX4 |
|  | RGS4 |
|  | RGS7 |
|  | RIMS1 |
|  | RNASE2 |
|  | RNF122 |
|  | RNF175 |
|  | ROBO2 |
|  | RRAGA |
|  | RRAGB |
|  | RRAS |
|  | RTN1 |
|  | RTN4IP1 |
|  | RUNDC1 |
|  | RWDD2A |
|  | SCCPDH |
|  | SCG2 |
|  | SCG3 |
|  | SCG5 |
|  | SCN3B |
|  | SCN8A |
|  | SEMA3A |
|  | SENP8 |
|  | SERPINH1 |
|  | SERPINI1 |
|  | SEZ6L |
|  | SH2D5 |
|  | SH3GL2 |
|  | SIDT1 |
|  | SLC16A1 |
|  | SLC16A14 |
|  | SLC17A6 |
|  | SLC22A6 |
|  | SLC22A9 |
|  | SLC25A12 |
|  | SLC25A14 |
|  | SLC25A4 |
|  | SLC2A13 |
|  | SLC30A3 |
|  | SLC32A1 |
|  | SLC39A10 |
|  | SLC7A4 |
|  | SLC9A6 |
|  | SLCO4A1 |
|  | SLIT1 |
|  | SLIT2 |
|  | SLITRK1 |
|  | SLITRK4 |
|  | SNAP25 |
|  | SNCA |
|  | SNX10 |
|  | SNX4 |
|  | SOCS3 |
|  | SOSTDC1 |
|  | SPATA7 |
|  | SPINT2 |
|  | SPOCK1 |
|  | SST |
|  | SSTR1 |
|  | ST8SIA3 |
|  | STAT4 |
|  | STEAP2 |
|  | STX1A |
|  | STXBP1 |
|  | STYK1 |
|  | SULT4A1 |
|  | SUSD1 |
|  | SVOP |
|  | SYN1 |
|  | SYN2 |
|  | SYNGR3 |
|  | SYNPR |
|  | SYP |
|  | SYT13 |
|  | SYT4 |
|  | TAC1 |
|  | TAGLN3 |
|  | TARBP1 |
|  | TC2N |
|  | TCL1B |
|  | TGFBR1 |
|  | THNSL1 |
|  | THY1 |
|  | TLN2 |
|  | TM4SF1 |
|  | TMEM130 |
|  | TMEM132D |
|  | TMEM151A |
|  | TMEM158 |
|  | TMEM169 |
|  | TMEM17 |
|  | TMOD1 |
|  | TMX4 |
|  | TNFRSF10B |
|  | TNS1 |
|  | TOLLIP |
|  | TRAPPC2L |
|  | TRIM36 |
|  | TRO |
|  | TSPAN7 |
|  | TUBA4A |
|  | TUBB2A |
|  | TXNIP |
|  | UBE2N |
|  | UBE2T |
|  | UCHL1 |
|  | UNC5B |
|  | VGF |
|  | VIP |
|  | VIPR1 |
|  | VRK1 |
|  | VSNL1 |
|  | VSTM1 |
|  | VSTM2A |
|  | VSTM2L |
|  | VTA1 |
|  | WBSCR17 |
|  | WDR54 |
|  | WIF1 |
|  | WNT10B |
|  | XK |
|  | XKR4 |
|  | YWHAB |
|  | YWHAZ |
|  | ZBTB20 |
|  | ZCCHC12 |
|  | ZDHHC23 |
|  | ZIC2 |
|  | ZMAT4 |
|  | ZNF215 |
|  | ZNF385B |
|  | ZNF391 |
|  | ZNF540 |
|  | ZNF652 |
|  | ZWILCH |
| Yellow | ACSS3 |
|  | AKR1C3 |
|  | ATOH7 |
|  | CA10 |
|  | CALB1 |
|  | CAMK4 |
|  | DDIT4L |
|  | FAT1 |
|  | FREM3 |
|  | GJA1 |
|  | GOLIM4 |
|  | GRAMD3 |
|  | ID4 |
|  | ITGA6 |
|  | MT1F |
|  | MT1X |
|  | MYO10 |
|  | NT5DC3 |
|  | PADI2 |
|  | PREX1 |
|  | PRKX |
|  | PTPRR |
|  | RFTN2 |
|  | SASH1 |
|  | SLC14A1 |
|  | SLC7A2 |
|  | SPHKAP |
|  | STON2 |
|  | STXBP5L |
|  | TLR4 |
|  | TOB1 |
|  | YAP1 |

**Supplementary Table 4. GSEA terms of five hub genes.**

| **Gene** | **Gene sets** | **Size** | **ES** | **NES** | ***P* value** | **FDR** |
| --- | --- | --- | --- | --- | --- | --- |
| AP3B2 | KEGG_LONG_TERM_POTENTIATION | 69 | 0.54 | 1.85 | 0.00 | 0.24 |
|  | KEGG_AMYOTROPHIC_LATERAL_SCLEROSIS_ALS | 52 | 0.49 | 1.79 | 0.01 | 0.21 |
|  | KEGG_CALCIUM_SIGNALING_PATHWAY | 176 | 0.46 | 1.77 | 0.00 | 0.17 |
|  | KEGG_AMINOACYL_TRNA_BIOSYNTHESIS | 41 | 0.55 | 1.77 | 0.02 | 0.13 |
|  | KEGG_CARDIAC_MUSCLE_CONTRACTION | 71 | 0.50 | 1.76 | 0.00 | 0.11 |
|  | KEGG_EPITHELIAL_CELL_SIGNALING_IN_HELICOBACTER_PYLORI_INFECTION | 66 | 0.40 | 1.70 | 0.01 | 0.16 |
|  | KEGG_ERBB_SIGNALING_PATHWAY | 85 | 0.41 | 1.68 | 0.01 | 0.16 |
|  | KEGG_PHOSPHATIDYLINOSITOL_SIGNALING_SYSTEM | 76 | 0.43 | 1.66 | 0.00 | 0.17 |
|  | KEGG_GNRH_SIGNALING_PATHWAY | 100 | 0.39 | 1.64 | 0.01 | 0.17 |
|  | KEGG_AXON_GUIDANCE | 127 | 0.43 | 1.62 | 0.01 | 0.19 |
|  | KEGG_NEUROTROPHIN_SIGNALING_PATHWAY | 125 | 0.35 | 1.62 | 0.01 | 0.17 |
|  | KEGG_TYPE_II_DIABETES_MELLITUS | 45 | 0.44 | 1.61 | 0.01 | 0.16 |
|  | KEGG_GLYCOSPHINGOLIPID_BIOSYNTHESIS_GANGLIO_SERIES | 15 | 0.65 | 1.57 | 0.01 | 0.21 |
|  | KEGG_GAP_JUNCTION | 84 | 0.44 | 1.54 | 0.04 | 0.23 |
|  | KEGG_OOCYTE_MEIOSIS | 109 | 0.36 | 1.53 | 0.02 | 0.24 |
|  | KEGG_ENDOCYTOSIS | 173 | 0.35 | 1.51 | 0.04 | 0.25 |
|  | KEGG_RIBOSOME | 86 | -0.52 | -1.96 | 0.01 | 0.02 |
| GABRD | KEGG_RIBOSOME | 86 | -0.50 | -1.90 | 0.02 | 0.09 |
|  | KEGG_NOTCH_SIGNALING_PATHWAY | 47 | -0.54 | -1.88 | 0.01 | 0.05 |
|  | KEGG_MATURITY_ONSET_DIABETES_OF_THE_YOUNG | 25 | -0.71 | -1.84 | 0.00 | 0.05 |
|  | KEGG_SMALL_CELL_LUNG_CANCER | 84 | -0.42 | -1.71 | 0.02 | 0.16 |
|  | KEGG_CHRONIC_MYELOID_LEUKEMIA | 72 | -0.41 | -1.69 | 0.02 | 0.16 |
|  | KEGG_PROSTATE_CANCER | 88 | -0.39 | -1.62 | 0.01 | 0.23 |
|  | KEGG_ANTIGEN_PROCESSING_AND_PRESENTATION | 87 | -0.50 | -1.56 | 0.03 | 0.24 |
|  | KEGG_ECM_RECEPTOR_INTERACTION | 83 | -0.47 | -1.56 | 0.03 | 0.23 |
|  | KEGG_ALLOGRAFT_REJECTION | 37 | -0.61 | -1.53 | 0.03 | 0.25 |
| GPR158 | KEGG_LONG_TERM_POTENTIATION | 69 | 0.50 | 1.73 | 0.03 | 0.21 |
| KIAA0513 | KEGG_RIBOSOME | 86 | -0.55 | -2.00 | 0.00 | 0.02 |
|  | KEGG_ANTIGEN_PROCESSING_AND_PRESENTATION | 87 | -0.58 | -1.84 | 0.00 | 0.08 |
|  | KEGG_GRAFT_VERSUS_HOST_DISEASE | 41 | -0.68 | -1.77 | 0.00 | 0.10 |
|  | KEGG_HEMATOPOIETIC_CELL_LINEAGE | 86 | -0.52 | -1.72 | 0.00 | 0.13 |
|  | KEGG_PHENYLALANINE_METABOLISM | 17 | -0.55 | -1.63 | 0.01 | 0.19 |
|  | KEGG_SMALL_CELL_LUNG_CANCER | 84 | -0.41 | -1.63 | 0.03 | 0.17 |
|  | KEGG_ALLOGRAFT_REJECTION | 37 | -0.63 | -1.60 | 0.02 | 0.20 |
| MAL2 | KEGG_AMINOACYL_TRNA_BIOSYNTHESIS | 41 | 0.65 | 1.97 | 0.00 | 0.05 |
|  | KEGG_OXIDATIVE_PHOSPHORYLATION | 99 | 0.57 | 1.95 | 0.01 | 0.03 |
|  | KEGG_PARKINSONS_DISEASE | 97 | 0.50 | 1.82 | 0.02 | 0.10 |
|  | KEGG_UBIQUITIN_MEDIATED_PROTEOLYSIS | 129 | 0.38 | 1.79 | 0.01 | 0.10 |
|  | KEGG_CARDIAC_MUSCLE_CONTRACTION | 71 | 0.52 | 1.79 | 0.00 | 0.08 |
|  | KEGG_ALZHEIMERS_DISEASE | 142 | 0.45 | 1.78 | 0.01 | 0.08 |
|  | KEGG_NEUROTROPHIN_SIGNALING_PATHWAY | 125 | 0.38 | 1.75 | 0.00 | 0.09 |
|  | KEGG_BASAL_TRANSCRIPTION_FACTORS | 35 | 0.53 | 1.74 | 0.00 | 0.09 |
|  | KEGG_ERBB_SIGNALING_PATHWAY | 85 | 0.43 | 1.73 | 0.00 | 0.09 |
|  | KEGG_AXON_GUIDANCE | 127 | 0.46 | 1.73 | 0.00 | 0.08 |
|  | KEGG_EPITHELIAL_CELL_SIGNALING_IN_HELICOBACTER_PYLORI_INFECTION | 66 | 0.41 | 1.72 | 0.02 | 0.08 |
|  | KEGG_LONG_TERM_POTENTIATION | 69 | 0.52 | 1.71 | 0.01 | 0.08 |
|  | KEGG_RNA_POLYMERASE | 29 | 0.47 | 1.69 | 0.02 | 0.09 |
|  | KEGG_PURINE_METABOLISM | 147 | 0.42 | 1.67 | 0.00 | 0.09 |
|  | KEGG_HUNTINGTONS_DISEASE | 158 | 0.41 | 1.66 | 0.03 | 0.10 |
|  | KEGG_AMYOTROPHIC_LATERAL_SCLEROSIS_ALS | 52 | 0.46 | 1.62 | 0.03 | 0.12 |
|  | KEGG_OOCYTE_MEIOSIS | 109 | 0.39 | 1.62 | 0.01 | 0.12 |
|  | KEGG_VIBRIO_CHOLERAE_INFECTION | 53 | 0.39 | 1.61 | 0.04 | 0.12 |
|  | KEGG_NITROGEN_METABOLISM | 23 | 0.60 | 1.58 | 0.03 | 0.14 |
|  | KEGG_PYRIMIDINE_METABOLISM | 95 | 0.40 | 1.58 | 0.01 | 0.13 |
|  | KEGG_RENAL_CELL_CARCINOMA | 66 | 0.37 | 1.55 | 0.05 | 0.16 |
|  | KEGG_GLYCOSPHINGOLIPID_BIOSYNTHESIS_GANGLIO_SERIES | 15 | 0.62 | 1.52 | 0.04 | 0.17 |
|  | KEGG_TYPE_II_DIABETES_MELLITUS | 45 | 0.42 | 1.52 | 0.03 | 0.17 |
|  | KEGG_CALCIUM_SIGNALING_PATHWAY | 176 | 0.39 | 1.50 | 0.02 | 0.17 |
|  | KEGG_SNARE_INTERACTIONS_IN_VESICULAR_TRANSPORT | 38 | 0.40 | 1.50 | 0.04 | 0.17 |
|  | KEGG_LONG_TERM_DEPRESSION | 66 | 0.41 | 1.50 | 0.02 | 0.17 |
|  | KEGG_PANTOTHENATE_AND_COA_BIOSYNTHESIS | 16 | 0.59 | 1.49 | 0.04 | 0.17 |
|  | KEGG_GLYCOSAMINOGLYCAN_BIOSYNTHESIS_HEPARAN_SULFATE | 26 | 0.55 | 1.47 | 0.04 | 0.18 |
|  | KEGG_PROGESTERONE_MEDIATED_OOCYTE_MATURATION | 84 | 0.34 | 1.44 | 0.04 | 0.20 |
|  | KEGG_MAPK_SIGNALING_PATHWAY | 260 | 0.34 | 1.43 | 0.04 | 0.19 |
|  | KEGG_T_CELL_RECEPTOR_SIGNALING_PATHWAY | 106 | 0.33 | 1.41 | 0.04 | 0.19 |
|  | KEGG_INSULIN_SIGNALING_PATHWAY | 136 | 0.29 | 1.40 | 0.05 | 0.19 |
|  | KEGG_FC_EPSILON_RI_SIGNALING_PATHWAY | 78 | 0.34 | 1.38 | 0.04 | 0.21 |

Note: ES, Enrichment Score; FDR, false discovery rate; GSEA, Gene Set Enrichment Analysis; NES, normalized ES. Only GSEA terms with *P* value < 0.05 and FDR < 0.25 were listed.
